# Individual Choices in Dynamic Networks: An Experiment on Social Preferences

**DOI:** 10.1371/journal.pone.0092276

**Published:** 2014-04-14

**Authors:** Dennie van Dolder, Vincent Buskens

**Affiliations:** 1 Erasmus School of Economics, Erasmus University Rotterdam, Rotterdam, the Netherlands; 2 Department of Sociology/ICS, Utrecht University, Utrecht, the Netherlands; 3 Erasmus School of Law, Erasmus University Rotterdam, Rotterdam, the Netherlands; University of Maribor, Slovenia

## Abstract

Game-theoretic models of network formation typically assume that people create relations so as to maximize their own outcome in the network. Recent experiments on network formation suggest that the assumption of self-interest might be unwarranted and that social preferences, such as altruism and inequality aversion, play a role in the formation of social networks. We developed an experiment to systematically investigate whether people show preferences for outcomes of others during network formation. We find that such preferences play a role when network decisions degenerate to simple two-person decision tasks. In more complex environments, however, we find little evidence for social preferences as a significant decision criterion. Furthermore, we find some evidence for farsighted behavior in network formation.

## Introduction

It has been shown that one's network position is not without consequences, having a crucial impact on many aspects of a person's life. Labor market outcomes [1], [2], job satisfaction [3], and health outcomes [4] are just a few examples of outcomes that are influenced by the network structure in which a person is embedded. Given that networks carry such weight and that people have some idea about the pattern of relations between others [5], it has been argued that people will try to maneuver themselves into optimal positions within the network [6], [7]. Empirical evidence supports this claim. People have been shown to evaluate the satisfaction with a relationship, costs of the relationship in terms of time and energy, quality of alternatives, past investments, and expected future of relationships when deciding on how much to invest in the relationship [8], [9]. Next to this, the assumption of purposive network formation can explain specific relational patterns found in the former GDR (East Germany) [10]–[12], and differences in investments in relationships between people in different European countries [13].

Given that people purposively build relations as to improve their position in a network, the development of game-theoretic models to capture this process seems a natural step [14], [15]. Broadly speaking, one can distinguish three lines of research applying game theory to social networks. First, one can distinguish papers that investigate how interacting on a fixed network structure influences actors' choices in specific games, such as prisoners' dilemmas or coordination games [16]–[19]. Second, one can distinguish papers that investigate which network structures emerge given particular payoff functions that map network positions to outcomes [14], [15], [20]. Finally, one can distinguish papers that study the co-evolution of network structure and behavior by studying what happens when actors play games on endogenous networks in which they can decide with whom to interact [21]–[24]. The current paper belongs to the second line of inquiry. We refer to Buskens et al. [25] for a more extensive overview of these lines of research and the distinctions between them.

Game-theoretic models of network formation make it possible to investigate stable networks depending on the relationship between the network structure and the actors' outcomes. Furthermore, by imposing a specific protocol by which relations are formed, they allow for an investigation of the formation process leading up to these stable states. At the level of the individual actor, game-theoretic models typically assume myopic self-interest. Myopic implies that actors only consider the direct payoff consequences of creating or removing a link. Self-interest implies that actors are assumed to only care about their own outcomes.

Experimental tests of these models indicate that they predict well when the outcomes are equal for all actors in the predicted network [26]–[28], but that the predicted networks are seldom observed if they provide unequal outcomes over actors [28], [29]. Furthermore, in the case where the networks formed coincide with the set of predicted networks, there appears to be a bias toward networks that maximize the sum of outcomes and networks in which everybody is equally well off [26]. In an experiment where subjects frequently formed a predicted network with highly unequal payoffs, they showed the unpredicted behavior of repeatedly rotating which of them had the most beneficial central position [30], [31]. Finally, it has been reported that individual network decisions are more likely if they increase equality in outcomes [28], [29].

Given that the protocol to form links and the payoff function used in such experiments closely resemble those applied to acquire theoretical results, the discrepancies reported above seem to be caused by incorrect assumptions regarding individual behavior. This suggest that, in terms of Coleman's schema of explanation [32], [33], assumptions regarding individual behavior at the micro level need to be improved to better understand the emergence of networks at the macro level. At the same time, the complexity of interdependencies that exists within networks makes it of crucial importance that we do not add too much complexity at the individual level in order to avoid that it becomes infeasible to derive macro-level implications [34]. Relaxing the assumption of self-interest is relatively straightforward and seems like a viable approach given that there is considerable evidence suggesting that people show pro-social behavior in various contexts [35]–[37]. Therefore, it may come as no surprise that the incorporation of social preferences, such as altruism and inequality aversion, into individual utility functions is the most frequently advocated remedy to increase predictive and explanatory power of the game-theoretic models [28]–[31].

Although the past results point in the direction of social preferences, explanations relying on rationality related arguments might also account for the findings reported above. In particular, one could relax the assumption of myopia instead of the assumption of self-interest. Due to the complexity of the interdependencies that exist within networks this option is, however, more involved. The few papers that have analyzed farsightedness in network formation show that for this extension it is not straightforward to derive predictions for individual behavior and related macro-level outcomes [38]–[40]. Still, this is no reason to rule out this approach a priori. Further research on individual level decision-making is required to determine which of the two approaches is most promising.

In the current paper we present an exploratory study to inform the debate on how game-theoretic models of network formation can best be extended at the micro level. We designed an experiment to investigate the importance of social preferences in the complex setting of network formation. In our experiment, subjects could choose their relations in continuous time in, for experimental settings, large groups of between 10 and 15 subjects. In designing our experiment, we focused on two types of social preferences that dominate the literature, namely: a concern for the absolute outcome of others, or altruism, and a concern for equality in outcomes. Subjects faced four different contexts in which contrasts between these preferences were likely to emerge. We obtained independent behavioral measures of their social preferences using the ring game proposed by Liebrand [41], and investigate whether behavior in the network formation experiment was related to these social preferences.

## Theory

### Social preferences

It has long been recognized that in order to understand situations of social interaction we must allow for the possibility that people do not only care about their own outcomes, but might consider others' outcomes as well. More formally stated, we should allow for the possibility that (some) people transform the objective outcome distribution over all people into a subjective utility [42]. Naturally, there exists a myriad of ways in which one can construct non-standard utility models that incorporate a concern for others' outcomes. Two such forms of non-standard utility dominate the literature. The first is a concern for the outcomes of others, or *altruism*, next to a concern for own outcomes. Empirical research indicates that many people show a tendency to care about the outcomes of others and that the degree to which they do so predicts their behavior in both experimental games [41], [43] and real life situations such as volunteer work, charitable giving, and the use of public transportation [44]–[47]. The second is a concern for *equality* in outcomes next to a concern for own outcomes. The concept of equality has received considerable attention in the psychological research on justice and equity [48]. More recently, equality arguments have also been applied to explain major patterns in data deriving from experiments in economics [49], [50].

Based on the dominant approaches in the literature, we focus on altruistic and equality concerns next to a concern for own outcomes. While these motives are typically studied in separate literatures, there is no intrinsic reason why people could not be sensitive to both sources of utility [51], [52]. Indeed, empirical evidence suggests that actors who are more altruistic also attach a higher value to equality [52]. Given these preferences, the utility function of a person *i* is depicted by equation (1).
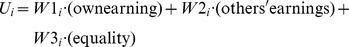
(1)


Where *W1_i_* represents the weight given by person *i* to own outcome, *W2_i_* is the weight given by person *i* to others' outcomes, and *W3_i_* is the weight given by person *i* to equality.

### Hypotheses

If people decide according to a concern for own outcomes (*W1_i_*) together with social preferences positing a concern for others' outcomes and equality respectively (*W2_i_* and *W3_i_*) we would expect some relations in a network to be more likely to be formed and maintained than others. On average, it can be assumed that *W1_i_*, *W2_i_*, and *W3_i_* are all positive. This is quite obvious for *W1_i_*. We expect that most, if not all, people prefer more for themselves compared to less holding others' outcomes and equality constant. For *W2_i_*, it has been shown that most people are classified as either caring positively about others' outcomes or not caring about them at all; only a small minority can be classified as caring negatively about others' outcomes [41], [43], [52]. Studies applying a continuous measure for *W2_i_* have shown the mean to be positive [52], . When it comes to *W3_i_*, theories on equality assume that people in general prefer equal distributions to unequal ones [49], [50], which is corroborated by empirical evidence [54]–[56]. Based on these arguments, we hypothesize that:

***H1.*** A subject is more likely to create or maintain a relation, the more this relation increases her own outcomes in the network.

***H2.*** A subject is more likely to create or maintain a relation, the more this relation increases others' outcomes in the network.

***H3.*** A subject is more likely to create or maintain a relation, the more this relation increases equality in the network.

One of the main points in the social preference literature is that not everybody has the same preferences. Multiple measurement methods have been proposed in order to assess social preferences at the individual level [43], [57]. Given that we can measure the weight that a person attaches to own outcome, others' outcomes, and equality independent of their decisions during network formation, we can construct the following hypotheses.

***H4.*** The more a subject cares about own outcome (i.e., the larger *W1_i_*), the larger the positive effect of own outcomes on the probability that this subject creates or maintains a relation.

***H5.*** The more a subject cares about others' outcomes (i.e., the larger *W2_i_*), the larger the positive effect of others' outcomes on the probability that this subject creates or maintains a relation.

***H6.*** The more a subject cares about equality (i.e., the larger *W3_i_*), the larger the positive effect of equality in outcomes on the probability that this subject creates or maintains a relation.

In order to test these hypotheses, we developed an experiment in which subjects interacted in several network formation games drawn from the game-theoretic network formation literature. In network formation, comparable to other game-theoretic models, the assumption of self-interest will be more problematic in some conditions than in others [58]. When the network formation process leads towards an efficient and equal network structure if actors behave according to self-interest, it is relatively unlikely that actors will face decisions that force them to weight off the importance they give to own outcomes, others' outcomes, and equality. Therefore, it might not matter a great deal whether actors are purely self-interested or not. When the formation process heads towards an inefficient and/or unequal network the preferences that actors have concerning others' outcomes and equality are more likely to play a role. Therefore, we imposed social contexts in which there was likely to be at least some tension between the aforementioned social preferences.

## Two specific models of network formation

In our experiment, we applied a truncated version of the connections model and the co-author model, both due to Jackson and Wolinsky [15]. In both models, relations are considered to be undirected and require consent of both actors involved. In other words, both actors *i* and *j* have to agree for the relation between *i* and *j* to form. We discuss the social context these models represent and how the different social preferences may come into conflict during network formation. The expected macro-level outcomes in these models receive relatively little attention here, but details can be found in Jackson and Wolinsky [15].

### The truncated connections model

Consider a situation in which the network is used to gain information. Actors receive (valuable) information from direct contacts and from actors that are at distance two in the network, where the distance between two actors is defined as the minimum number of links that have to be passed to get from one actor to the other. Actors do not receive any valuable information from others at a distance larger than two. Relations are costly because time and energy have to be invested in order to maintain a relationship. Under these assumptions, the outcome an actor receives from a specific network position can be represented by a truncated version of the connections model [15]. We denote the number of direct contacts of actor *i* with *n_i_*, and the number of actors at distance two with *m_i_*. We set the value of a direct contact to *α*, the value of an indirect contact at distance two to *β*, and the cost of a relation to be *c*. If a relation is formed, both actors have to pay a cost *c*. The outcome *p_i_*(*g*) of actor *i* is given by equation (2).
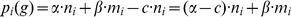
(2)


**Figure 1:**
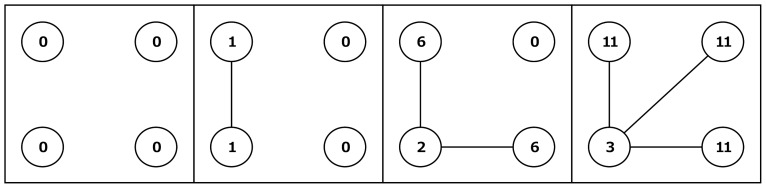
Examples of outcomes for the *CONLOW* condition. Shown are a number of outcome examples for the *CONLOW* condition that we employed in our experiment. Actors are depicted as circles; outcomes for each actor are denoted within these circles.

For the first experimental condition, we applied *β>α* − *c*>0 (specifically: *α* − *c*  =  1 and *β* = 5). We term this condition *CONLOW*, where “low” indicates the relatively low cost for maintaining relations compared to the other condition that we applied. Under this condition, a self-interested actor prefers to connect to actors who have many relationships. By connecting to an actor with many relations, one gains valuable indirect contacts. Such behavior is also beneficial for the group as a whole; the highly centralized star network (in which one actor is related to all others and there are no further relationships) maximizes the sum of outcomes [15]. Equality, however, decreases by such behavior as the central actors bear the cost for all their relationships, making them worse off than those in the periphery. Figure 1 illustrates how a process in which everybody connects to the most connected actor can lead to a highly unequal network. Even though the actor in the central position is worse off than the others, each relation still has a positive value to her (*α* − *c*  =  1). Assuming myopic self-interest, the central actor should thus be willing to create these relations. If actors value equality, this process might develop differently because inequality aversion will deter actors from forming star like structures. If the central actor in the star dislikes inequality, she will at some point refrain from giving consent for relationships to form. This is the case because she only benefits marginally from each additional relationship, while inequality raises steeply the more centralized the network becomes.

**Figure 2:**
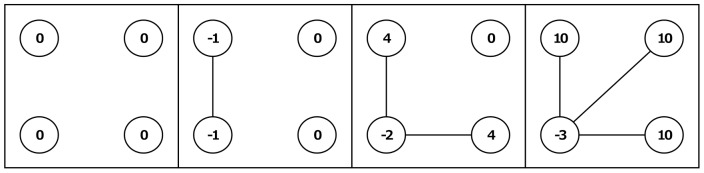
Example of outcomes for the *CONHIGH* condition. Shown are a number of outcome examples for the *CONHIGH* condition that we employed in our experiment. Actors are depicted as circles; outcomes for each actor are denoted within these circles.

The second condition that we applied in the experiment was *β>*0>*α* − *c* (specifically: *α* − *c  = * −1 and *β = *5). We term this condition *CONHIGH*. (To avoid that subjects in the experiment reached negative earnings too easily we increased their baseline outcome by 5 points.) To see the difference with the *CONLOW* condition, Figure 2 shows the outcomes of networks depicted in Figure 1 in the *CONHIGH* condition. A self-interested actor still wants to connect to actors who have many connections in order to maximize the number of indirect contacts. Now, however, actors no longer benefit from direct connections and (assuming myopic self-interest) highly centralized star structures become impossible. Therefore, the conflict between own outcomes and equality decreases. On the other hand, a conflict between own outcomes and altruism may arise. If an actor is altruistic she may be willing to take a more central position, even though this causes negative own outcomes and pronounced inequality. It should be noted that in this setting it takes some farsightedness or some degree of error for the formation process to get underway if we start from an empty network, because based on any of the hypothesized preferences no myopic actor has an incentive to create the first relation in the empty network. Thus, the empty network is stable. Other stable networks are networks in which each actor has at least two relations, and are thus characterized by circle shaped structures [15].

### The co-author model

The co-author model describes the benefits of researchers who co-author papers [15]. Opposite to the connections model, it deals with negative externalities. The basic intuition is that a researcher's time to spend on a project is inversely related to the number of projects she is working on. It is advantageous for a researcher to work on many projects. Researchers, however, prefer that their co-authors have as few projects as possible, because more projects make them less productive in each separate project. The outcome *p_i_*(*g*) of researcher *i* is given by equation (3) if *n_i_*>0 and *p_i_*(*g*) = 0 if *n_i_* = 0.
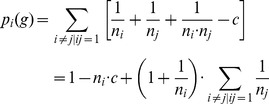
(3)


Here, *ij* indicates that there is a relation between actors *i* and *j*, *n_i_* represents the number of projects that *i* is involved in, *n_j_* represents the number of projects that *j* is involved in, and *c* represents an additional cost term for the maintenance of a relationship. (To make the earnings that could be obtained in this setting comparable to those in the connections model we multiplied the function by 20.)

**Figure 3:**
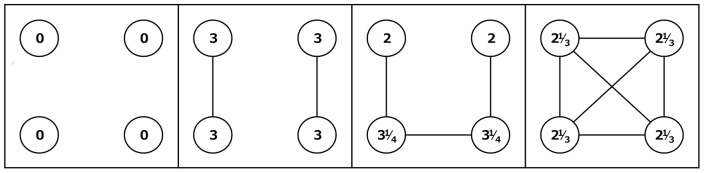
Example of outcomes for the *COALOW* condition. Shown are a number of outcome examples for the *COALOW* condition that we employed in our experiment. Actors are depicted as circles; outcomes for each actor are denoted within these circles.

In the first setting, we set *c* = 0. This condition, which we term *COALOW*, corresponds to the original co-author model of Jackson and Wolinsky [15]. The network situation can be characterized as a social dilemma in which every actor has an incentive to build as many relations as possible, but the social optimum would be to form mutually exclusive dyads. Because each actor has incentives to create many relations, the situation easily cascades towards the complete network, making everybody worse off in the long run. An example is shown in Figure 3. When everybody has at least one relationship, adding additional relations in the network decreases others' outcomes faster than it increases own outcome. If actors are altruistic or inequality averse, this might facilitate the formation of mutually exclusive dyads, and thereby resolve the social dilemma.

**Figure 4:**
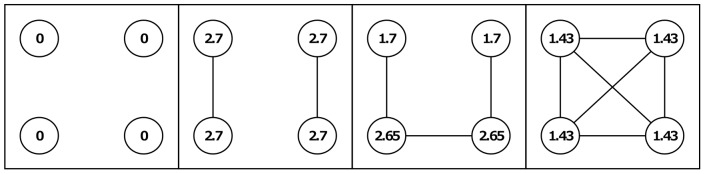
Example of outcomes for the *COAHIGH* condition. Shown are a number of outcome examples for the *COAHIGH* condition that we employed in our experiment. Actors are depicted as circles; outcomes for each actor are denoted within these circles.

In addition, we set *c* = 0.3: *COAHIGH*. To see the difference with the *COALOW* condition, Figure 4 shows the outcomes of networks depicted in Figure 3 in the *COAHIGH* condition. In this case, it is much more likely that actors stay in mutually exclusive dyads: if this situation is reached, no actor has an incentive to add or remove relations. Simulations in which actors were allowed to add and remove relations in a random order indicated that it is very unlikely that people actually reach dyads in large groups. Furthermore, if we assume a self-interested actor, only a small degree of error is needed in order for her to create an additional relationship, given that the costs incurred by such a deviation are relatively small. If an actor is altruistic or inequality averse, however, such mistakes are much less likely as the resulting drop in others' outcomes and equality will have a negative impact on the actor's utility.

## Experiment

### Participants

In total we ran 16 experimental sessions, each of them having between 9 and 15 subjects. Subjects were contacted using the Online Recruitment System for Economic Experiments [59] to participate in a study called “Let's Connect”. They were offered on average €16 but were informed that the exact amount would depend on their own and others' decisions. A total of 227 subjects subscribed for one of the 16 sessions, of which 205 subjects participated. We allowed at most 16 registrations for each session and every registered subject who showed up in time participated. Most subjects were students at Utrecht University from a wide range of disciplines, although non-student subjects also participated. Subjects were between 17 and 60 (mean age being 21.6), 68.3% female, and 78.5% Dutch.

All participants in the experiment had previously provided written consent when signing up online to participate in laboratory experiments at the ELSE laboratory in Utrecht. In doing so, they had indicated to having read and agreed to the rules regarding participation and proper laboratory behavior and the researchers' commitments and privacy policy. They were also informed that they could stop participating in the experiment whenever they wanted. While five of the subjects where 17 years of age, these were all healthy adult students and no subjects participated who could seriously be considered minors. Hence, no further consent from parents or caretakers was obtained.

All data were analyzed anonymously. The nature of this behavioral experiment, not involving any medical procedure and not obliging subjects to perform certain acts or behavior, does not require formal medical ethical approval according to the Dutch law [60]. This was confirmed by the Advisory Committee under the Medical Research (Human Subjects) Act of the Faculty of Social and Behavioural Sciences at Utrecht University.

### Procedure

Sessions were conducted in the ELSE laboratory at the Department of Sociology at Utrecht University. Subjects were assigned randomly to cubicles and received printed instructions in the language of their preference, either English or Dutch. The instructions started by welcoming them to the experiment, stating that they could ask questions at any time, that they could earn points during the experiment, and that 100 points equaled 1 Euro to be paid at the end of the experiment. After this, the instructions explained the various tasks employed in the experiment.

The experiment was conducted using the z-Tree computer software package for readymade economic experiments [61]. All subjects participated for three network formation rounds in each of the four conditions, implying that they played twelve rounds of network formation overall. The first round in each condition was a trial round lasting one and a half minute. This allowed subjects to gain experience with the particular network formation condition without it influencing their actual monetary outcomes. After this, they played two “real” rounds, each lasting five minutes, which did affect their monetary outcomes. The ordering of conditions differed between sessions to counterbalance learning between conditions. In the results section, we investigate whether the ordering of conditions in the experiment influences the results.

After completing the network formation task, subjects were presented with the so-called *ring game* in order to obtain measures of social preferences [41], [43], [62] and were administered a small questionnaire. When finished, the outcomes from the network formation game and the ring game were added together, communicated to the subjects, and the subjects received their earnings in private. The entire process lasted about one and a half hour and subjects on average received €20.70. Complete instructions for the experiment are available as an electronic supplement.

### Network formation task

We designed the network formation task in order to maximize possibilities for analyzing individual decisions. First, we allowed subjects to change their relations in *continuous time* with complete knowledge of what others were doing. Second, we calculated outcomes in continuous time and continuously updated the information relating to the outcomes on the screen. Since we are interested in individual decisions, we wanted to ensure *direct* incentives attached to changes in relationships. Third, we allowed subjects to form networks in larger groups than are typically employed in experiments on network formation. The reason for this is twofold. First, it allows for observing more individual decisions than one would observe in smaller networks. Second, such a setting is one step closer to a real-world sociological setting than a network experiment on very small (4 or 6 person) networks.

**Figure 5:**
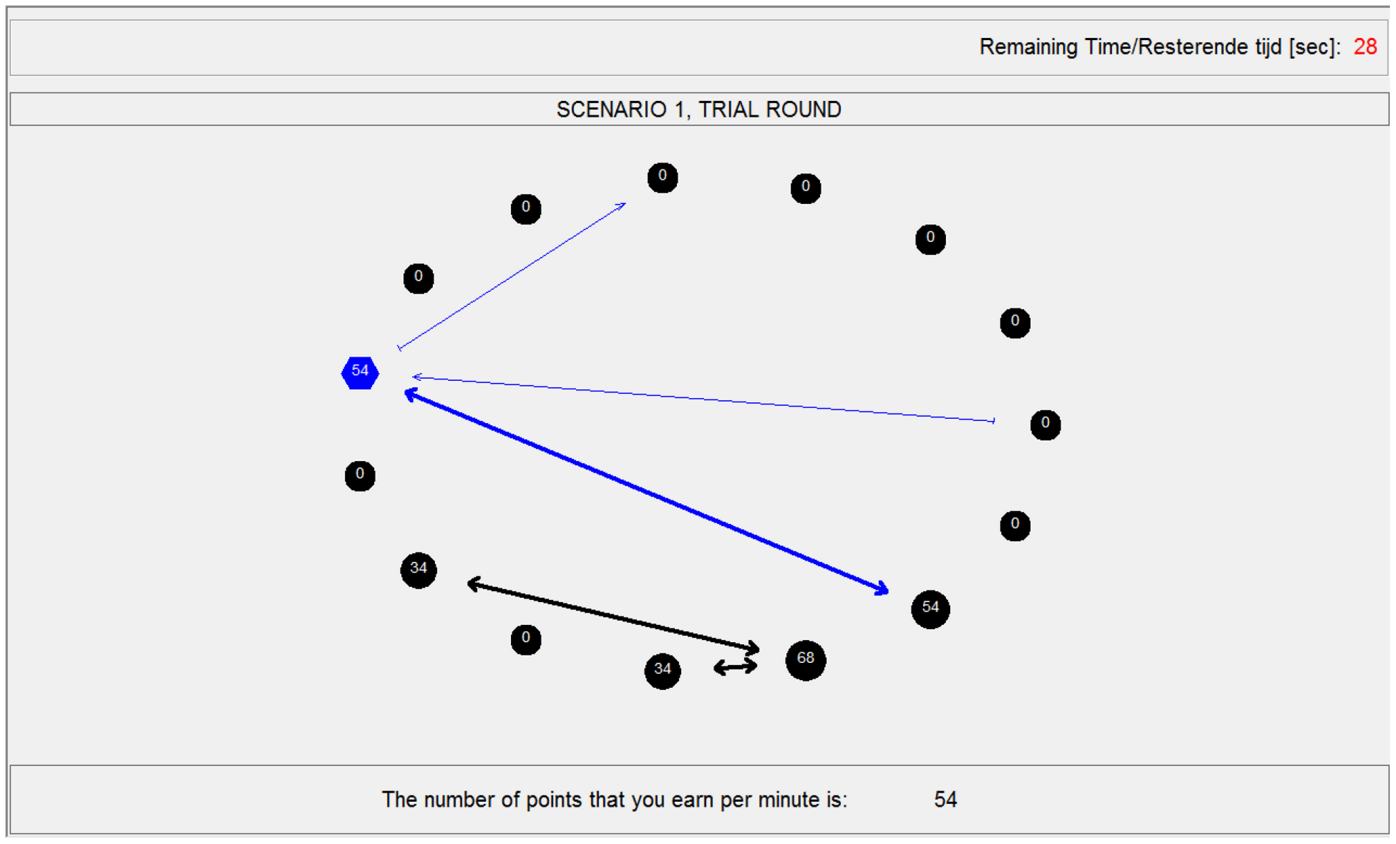
Screenshot of the experiment. The figure depicts the screen that was shown during the network formation experiment. This particular example is taken from the *COAHIGH* condition. The subject saw herself depicted as a (blue) hexagon, while she saw the others depicted as (black circles). Proposals for relations, which did not influence outcomes, were depicted as blue, thin, directed arrows from the given subject making the proposal to the other subject. Proposals were only visible to the two subjects involved. Relations where depicted as thick, double-headed arrows, colored blue on the screens of the subjects involved in the relation and black on the screens of the other subjects. In the upper right corner of the screen, the amount of seconds left in the current network formation round was shown. In a bar above the subjects, the scenario and round were stated. After each round, subjects were reshuffled on the screen, ensuring anonymity between rounds. The outcomes per minute for the subject were shown at the bottom of her screen and in the blue hexagon. The outcomes for the other subjects were shown in the black circles. Next to this, the size of both the hexagon and the circles changed with the number of points that the subjects earned: larger in size meaning that the particular subject earned more points per minute.

In the network formation task, all subjects were depicted on the computer screen (Figure 5). Each subject saw herself depicted as a (blue) hexagon, while she saw the others depicted as (black) circles. This allowed subjects to clearly distinguish between themselves and the other subjects. A subject could propose a relation by clicking on another subject's circle and could withdraw an existing proposal by repeating this action. A proposal had no effect on outcomes; it merely provided a way in which a given subject could show another subject her interest in forming a relation. Proposals were depicted as a blue, thin, directed arrow from the subject making the proposal to the other subject (see top two arrows in Figure 5). Because proposals did not matter in determining actual network positions and outcomes, a proposal was only visible for the two subjects involved. Given that a proposal existed, it was possible for a relation to form. After a subject made a proposal for a relation with another subject, this other subject could create the relation by clicking on the former subject. A relation was depicted as a thick, double-headed arrow, colored blue on the screens of the subjects involved in the relation and black on the screens of the other subjects. Once a relationship was formed, either subject could remove it by simply clicking on the other subject.

The amount of seconds left in the current network formation round was shown in the upper right corner of the screen. The scenario and round were stated in a bar above the network and corresponded with the explanations of the specific conditions in the instructions. This information allowed subjects to easily locate where they were in the experiment at any point in time (see Figure 5). After each round, subjects were reshuffled on the screen, ensuring anonymity between rounds.

The screen also provided insights in what subjects earned. While outcomes were calculated per second they were shown per minute because the outcomes per second were very low. Subjects were clearly explained that outcomes were calculated per second and that if, for example, they would earn 90 points per minute for 10 seconds they would receive 10/60 times 90  =  15 points for these 10 seconds. The outcomes per minute for a given subject were shown at the bottom of the screen and in the blue hexagon. The outcomes for the other subjects were shown in the black circles. Next to this, the size of both the hexagon and the circles changed with the number of points that the subjects earned: larger in size meaning that the particular subject earned more points per minute. These shifts in sizes were made to allow subjects to take the outcomes for others into account in a more intuitive way than looking purely at numerical values. The subjects only saw what they and the others were earning individually at that point in time and did not see any aggregate measures on the sum of outcomes for the group or the equality of outcomes.

### Ring game

To test the hypotheses on differences between individuals in the likelihood to create or maintain a relation, we needed to obtain independent measures of their social preferences. We used the *ring game* [41], [43], [62] to acquire such measurements.

**Figure 6:**
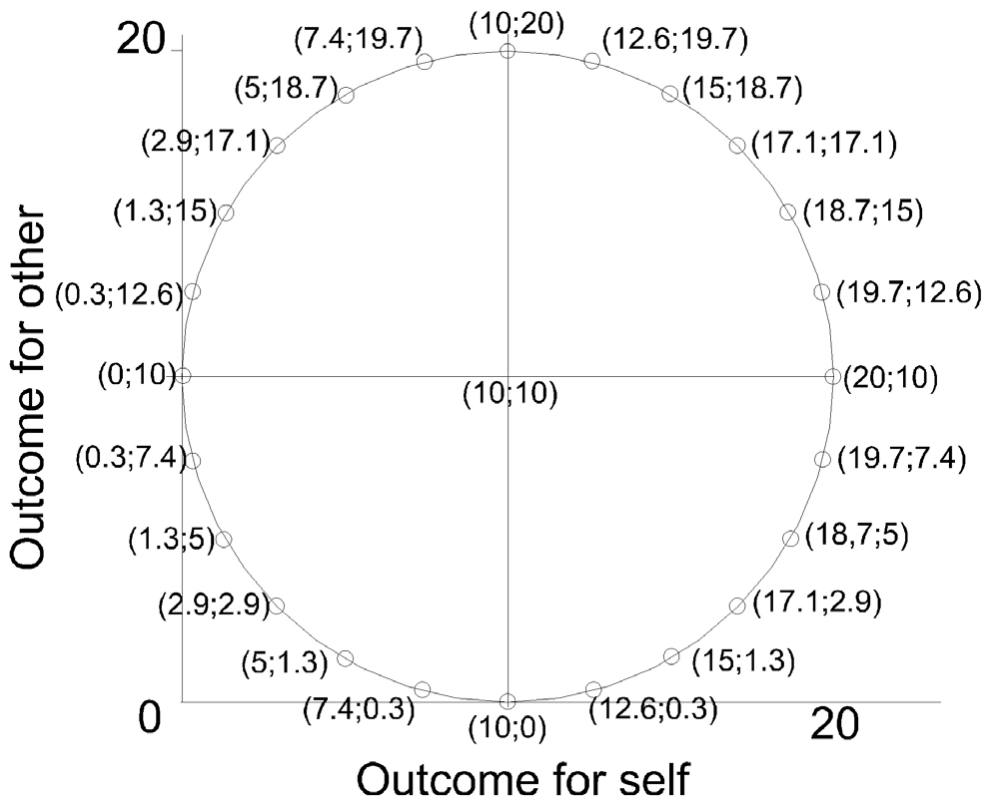
The own-other outcome circle used for the ring game. The plane is defined by two orthogonal dimensions representing own and other's outcomes. The center of the ring was placed at the (10,10) coordinate, with a radius of 10. Subjects had to make 24 choices between two equidistant own-other outcome distributions located next to each other on the circle.

In the ring game, 24 pairs of own-other outcome sets are selected from a circle in the own-other outcome plane. This plane is defined by two orthogonal dimensions representing own and other's outcomes. Each of the 24 decisions concerns a decision between two equidistant own-other outcome distributions located next to each other on the circle. While the center is typically placed at the origin, we displaced the center to the (10, 10) coordinate, and set the radius to be 10, displayed in Figure 6. Subjects were, for example, asked to choose whether they preferred 10 for themselves and 20 for the other or 12.6 for themselves and 19.7 for the other. Earnings awarded by the choices of other subjects remained concealed until the 24 choices were completed. We chose to avoid negative outcomes, because such outcomes were either infeasible (*CONLOW*, *COALOW*) or unlikely (*CONHIGH*, *COAHIGH*) in the network formation games. Next to this, distributions of only positive outcomes are considered to be easier to evaluate for subjects, and more useful in eliciting preferences concerning equality [52].

## Methods

### Analysis strategy

The network formation experiment generated a large amount of data. In total, the 205 subjects in our experiment made 117,715 decisions (clicks on other subjects). We neglect all decisions made in the trial rounds and all decisions to create or remove one-sided proposals for relations, because these decisions did not influence actual outcomes. After excluding these cases, there are 67,917 decisions left.

In order to be able to analyze these data statistically, we need to impose some assumptions. Throughout the analyses and in line with the dominant theoretical approach, we will assume that subjects are myopic. That is, we assume that subjects purely consider the current outcomes resulting from a relation when deciding to create or maintain it.

We reorganize the data into a series of *pairwise comparisons*. In particular, we assume that if a change is made in the network, the subject involved evaluates the situation with and without the relation and acts according to her preferences. If a change leads to an undesirable result, it can be reversed immediately. Naturally, such a reversal must be made within a short time period after the initial change. If a subject removes a relation multiple seconds or even minutes after the initial creation, this decision cannot reasonably be interpreted as the result of a pairwise comparison of the situation with and without the relation. We, therefore, analyze whether or not a change is reversed *within a short evaluation period after the initial change*. Time is measured in discrete seconds, so we choose the period in which a change could be reversed as either being the second in which the change is made or the consecutive second. In the results section, we investigate whether increasing the length of the evaluation moment influences our results.

When a subject removes a relation, the pairwise comparison process and its interpretation are quite straightforward. If a subject removes a relation and does not reverse this decision, this indicates that this subject prefers the situation without the relation to the situation with the relation. If a subject removes a relation and reverses this decision, this indicates that the subject prefers the situation with the relation to the situation without the relation. We omit decision situations in which the other subject removes the proposal within the evaluation period following the removal of the link. In these cases the original subject no longer has the opportunity to recreate the link.

When a subject creates a relation, both subjects involved in the relation can decide to reverse it because mutual consent is needed. If the creation of a relation is reversed, this indicates that the subject that reversed the relation prefers the situation without the relation to the situation with the relation. If the relation is maintained, however, this signals that both subjects involved in the relation prefer the situation with the relation to the situation without the relation. In the model we cannot add variables for both subjects because in all other cases there is only one subject who makes the decision. Therefore, we have to combine the variables over the two subjects or select one subject as the crucial decision maker. We decide to take the subject who initiated the change as the crucial decision maker, since this subject is most likely to actively evaluate the result of the initial change. In the results section, we investigate the robustness of our results with regards to this assumption.

### Analysis method

In our analyses, we apply the Thurstone-Mosteller model for pairwise comparisons [63]–[68]. We assume that a subject assigns a utility *U* to the situation with and the situation without the relation, shown in equations (4) and (5) below.
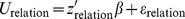
(4)

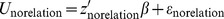
(5)


Here *z* may include both attributes of subjects themselves and specific aspects of the outcome distribution. In this function *z*'*β* represents the deterministic component of the utility function, and *ε* represents the random component that can be interpreted as the part of the utility that cannot be explained by the deterministic function.

The probability that the situation with a relation is chosen over the situation without a relation can then be written as shown in equation (6).
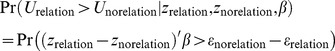
(6)


If we assume that, apart from the hierarchical nesting for which we control in the analysis, the random terms *ε* are independently and identically distributed with the type I extreme-value distribution (see page 59 of Maddala [69]), the probability that a subject chooses having the relation over not having the relation is given by equation (7).
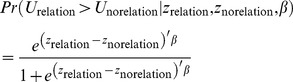
(7)


Equation (7) corresponds to a binary logit model in which the independent variables are the differences between the two options. Because the independent variables are differences between the two situations (i.e., with and without the relation), effects for variables that do not vary between these situations (such as personal characteristics) are not identified. Still, we can use such variables to construct interaction effects.

We run separate analyses for each of the four conditions. Also, we take into account that the observations are nested. Decisions are nested within directed dyads, which are nested within decision makers, which are nested within sessions. In order to take this into account we run hierarchical four-level logistic regression models in which we estimate random intercepts at each level [70]. By focusing on one decision maker, we neglect some dependence between observations. In particular, we neglect dependence related to the other subject in the dyad. We ran several other models, e.g., taking the other actor in the dyad as the decision maker as well as non-hierarchical versions with random effects for both subjects. All these models led to similar coefficients and significance levels. Moreover, other random effects are small in almost all other models. This provides confidence that the most important random effects are incorporated.

### Network variables

The dependent variable is whether (1) or not (0) a relation exists after an evaluation moment. The first independent variable comprises the outcome for the decision maker. For altruism, we look at the sum of others' outcomes. Denoting the outcome for subject *i* in the situation with the relationship under evaluation by 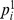 and the outcome for subject *i* in the situation without the relation under evaluation by 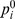, we compute the outcome variables as follows:
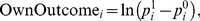
(8)

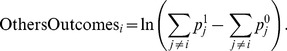
(9)


As can be seen in equations (8) and (9), we compute the natural logarithm of the differences between the situation with and without the relation for own outcome and others' outcomes, because the distributions of these variables have long and thin tails.

Finally, we operationalize equality as −1 times the standard deviation in outcomes in the group. The standard deviation provides a measure for inequality, and reversing it thus provides a measure for equality. Denoting the average outcomes over all subjects in the situation with the relation under evaluation by 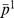, and the average outcomes over all subjects in the situation without the relation under evaluation by 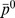, we compute the equality variable as follows:
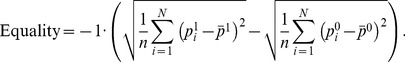
(10)


We use the standard deviation because it has some naturally appealing qualities. First, it does not heavily rely on the outcome of the decision maker. Second, the standard deviation increases more sharply if differences become higher. While in some cases this is a drawback, because it makes the standard deviation sensitive to outliers, we believe it is a positive aspect in the current study. It seems likely that subjects will not care too much about small differences in outcomes, independent of their social preferences, but these preferences will be increasingly important if inequality increases. We have also conducted our analyses using the Fehr and Schmidt's model on inequality aversion [50]. Fehr and Schmidt model a form of self-centered inequality aversion, by assuming that individuals put a positive weight on the outcomes of those that earn less than them and a negative weight on the outcomes of those that earn more than them. These analyses led to similar conclusions as the analyses presented here.

In the main analyses, we will assume that subjects care about the outcomes of all others. Alternatively, it is plausible that subjects focus on only a subset of others. In subsequent robustness analyses, we will vary the definition of the reference group and investigate whether this influences our results. In particular, we will allow subjects to care only about the outcomes of those with whom they have a relationship or, even more narrowly, only about the specific other with whom they are currently considering a relationship.

### Social preference measurements

We use the 24 decisions that subjects made in the *ring game* to measure their social preferences. Van Lange [52] introduced a simple method to determine the degree to which a subject's decisions are influenced by her own outcomes (*W1_i_*), others' outcomes (*W2_i_*), and equality (*W3_i_*). The total outcome over 24 decisions that a subject allocated to herself and to the other can vary between 220 and 260. The actual amount allocated is translated into a weight ranging between −1.00 and 1.00. If a subject allocated *x* to the others, the weight attached to the others' outcome is (*x* – 240)/20. The weight assigned to own outcomes is calculated exactly the same. For the weight attached to equality, we calculate the sum of the absolute difference between own and others' outcome over the 24 decisions. The minimum here was 186.56, the maximum was 243.13. Just as above, the weight attached to equality was rescaled between 1.00 (if the subject minimized inequality at 186.56) to −1.00 (if the subject maximized inequality at 243.13). We have also conducted analyses in which we estimated the subjects' social preferences towards altruism and inequality by means of logit analyses. These estimates correlate very highly with the estimates derived here (Pearson coefficient over 0.85). Therefore, we use these relatively simple ways to compute the estimates.

## Results

### Social preferences

Before we proceed to the findings deriving from the network experiments, we briefly discuss the outcome of the social preference measurements, as these measurements serve as inputs for the subsequent analyses. We find considerable variation in each of these variables. Furthermore, the means of all three variables are positive, although not to the degree that one may have expected. The value given to own outcome varied between 0 and 1, with a mean of 0.83 and a standard deviation of 0.20. The value given to other's outcome varied between −0.79 and 1, with a mean of 0.12 and a standard deviation of 0.29. Finally, the value given to equality varied between −0.34 and 0.59, with a mean of 0.04 and a standard deviation of 0.16.

### Results for the connections model

We start by analyzing the connections model with low cost (*CONLOW*). The results are shown in Table 1. Model 1a is the baseline model, only including a variable indicating whether the initial change was to create a relation (1) or delete a relation (0), and a constant. Recall that the dependent variable is whether or not a relation exists after an evaluation moment. The idea is that subjects compare the situation with and without a relation and choose the situation they prefer. For whatever reason, it might be that subjects have a tendency to stick to the initial decision to either create or remove a relation. Therefore, we add the “creation of relation” dummy and the constant. The constant relates to the likelihood that a subject recreates a relation if the initial decision was to delete it, the sum of the “creation of a relation” effect and the constant relates to the likelihood that a subject maintains a relation if the initial decision was to create it. In Models 1a and 1b, we see that subjects indeed have a tendency to stick to the initial decision. The constant is negative indicating that if the subject initially removed a relation, there is a tendency not to recreate it. The “creation of relation” effect is positive and larger than the constant, indicating that if a relation is created subjects have a tendency to maintain it. These effects are relatively stable over analyses, and since they do not pertain to the hypotheses, they will not receive further attention.

**Table 1:** Logistic regression results for the *CONLOW* and *CONHIGH* conditions.

	CONLOW				CONHIGH			
	Model 1a		Model 2a		Model 1b		Model 2b	
*Fixed effects*								
Constant	−0.769	(0.000)	−0.478	(0.000)	−1.157	(0.000)	−0.807	(0.000)
Creation of relation	1.938	(0.000)	1.982	(0.000)	2.043	(0.000)	2.078	(0.000)
Own outcome			3.806	(0.000)			2.917	(0.000)
Others' outcomes			0.242	(0.011)			−0.172	(0.050)
Equality			−0.202	(0.000)			−0.165	(0.000)
*W1* * own outcome			0.986	(0.001)			0.729	(0.001)
*W2* * others' outcomes			−0.941	(0.001)			−0.072	(0.767)
*W3* * equality			0.060	(0.691)			0.037	(0.792)
*Random effects*								
Session	0.088		0.086		0.003		0.000	
Decision maker	0.097		0.264		0.321		0.348	
Directed dyad	1.074		0.589		0.869		0.569	
Number of sessions	16		16		16		16	
Number of decision makers	205		205		205		205	
Number of directed dyads	3778		3778		3672		3672	
Number of decisions	21208		21208		19997		19997	
Log likelihood	−12317.31		−9903.68		−11525.27		−9396.76	

The table shows the hierarchical four-level logistic regression estimates on whether (1) or not (0) a relation is present after an evaluation moment in the *CONLOW* and the *CONHIGH* conditions. *Creation of relation* is a dummy variable indicating whether the initial change was to create a relationship (1) or remove a relationship (0). *Own outcome* denotes the natural logarithm of the difference in own outcome between the situation with and without the relationship. Others' outcome denotes the natural logarithm of the difference in others' outcomes between the situation with and without the relationship. *Equality* denotes the difference in equality between the situation with and without the relationship. W1 * own outcome, W2 * others' outcomes, and W3 * equality denote the interaction effects between the aforementioned variables and the associated social preference measurements derived in the ring game. Both outcome variables and measures of social preference are centered around their respective means. Random effects are estimated at the level of the session, the decision maker, and the directed dyad under consideration. Numbers of sessions, decision makers, directed dyads, and decisions are given, as are the log likelihoods. *P*-values are in parentheses.

In Table 1, Model 2a, we add the variables for own outcome, others' outcomes, and equality, as well as the interaction effects of the aforementioned variables with the associated social preference measurements derived from the ring game. Note that the social preferences are subject characteristics and that, therefore, their main effects are not identified in the pairwise comparison analyses. We center all variables around their means in order to facilitate interpretation of the constant and the main effects of outcome variables. We find that subjects are more likely to choose for relations, the more these relations increase their own outcomes and the more these relationships increase others' outcomes. Contrary to the predictions, equality has a significant negative effect suggesting that the more a relation increases equality in the network, the less likely it is to be created or maintained.

As shown in Model 2a, subjects with a higher value for their own outcome in the ring game give significantly more weight to their own outcomes in network formation. Surprisingly, we find that the value a subject assigns to the other's outcome according to the ring game has a significant negative effect on the weight given to others' outcomes during network formation. This indicates that subjects who attach a high value to the outcome of the other subject in the ring game are less likely to create or maintain relations that increase others' outcomes. The interaction relating to equality is not statistically significant.

Models 1b and 2b in Table 1 show the analyses for the connections model with high cost (*CONHIGH*). As in the *CONLOW* case, subjects tend to create and maintain relations that increase their own outcome and decrease the equality in the network. Contrary to the *CONLOW* case, the main effect of others' outcomes is negative; subjects are more likely to create or maintain links that lower others' outcomes. With regard to the interactions, we now find that the importance given to own outcomes as measured in the ring game is the only preference that significantly influences decisions. Subjects who give more weight to own outcomes in the ring game give more weight to own outcomes in the decision task. The other two interactions effects are not significant. A noteworthy finding, not shown in the tables, is that for both *CONHIGH* and *CONLOW*, almost the entire improvement in model fit between Models 1 and 2 comes from adding main effects; the interactions explain little variance in the data.

In short, Hypothesis 1 is supported: subjects are more likely to have relations that increase their own outcomes. Hypothesis 2 is not uniformly supported: while in the *CONLOW* condition subjects are more likely to have relations that increase others' outcomes, the opposite holds true in the *CONHIGH* condition. Hypothesis 3 is refuted, since subjects are significantly more likely to have relations that increase inequality. When looking at the interaction effects, we find that Hypothesis 4 is supported: subjects who act more in line with self-interest in the ring game also act more in line with self-interest in the network formation experiment. With regard to Hypotheses 5 and 6, the results are not supportive, and in the case of others' outcomes we even find contradictory evidence in the *CONLOW* condition. The lack of consistent support for the hypotheses relating to others' outcomes and equality, both in terms of the main effect and in terms of the interactions, questions whether our hypothesized mechanisms are at work in these contexts. We elaborate on this issue below.

### Results for the co-author model

Now we turn to the co-author model with low costs (*COALOW*). While analyzing the co-author model we dropped the cases in which someone connected to an isolate, because connecting to an isolate increases own outcomes, others' outcomes, and in many cases also equality. Straightforward calculations show that the change in own outcome equals the change in others' outcomes exactly if one connects to an isolate. These decisions create high correlations between the variables of interest, which causes problems in the statistical analyses.

Model 2c in Table 2 shows positive effects of own outcomes and others' outcomes, and a negative effect of equality. Again, we find that a larger concern for own outcomes in the ring game relates to a larger concern for own outcomes in network formation. For equality, we find that those caring more strongly about equality in the ring game also do so during network formation, while for others' outcomes we find no such relation. If we analyze the co-author model with high costs (*COAHIGH*), we find that the main effects are positive for own outcome and others' outcomes and insignificant for equality. With regard to the interaction terms, we find that a significant positive effect for others' outcomes; those caring more for others' outcomes in the ring game also do so during network formation. Furthermore, those who care more about equality in the ring game, care less about equality during network formation. As with the connections models discussed previously, and not visible in the tables, it is worth noting that almost the entire improvement in model fit between Models 1 and 2 comes from adding main effects, while interactions explain little variance in the data.

**Table 2:** Logistic regression results for the *COALOW* and *COAHIGH* conditions.

	COALOW				COAHIGH			
	Model 1c		Model 2c		Model 1d		Model 2d	
*Fixed effects*								
Constant	−0.476	(0.000)	−0.990	(0.000)	−1.588	(0.000)	−1.035	(0.000)
Creation of relation	3.130	(0.000)	3.173	(0.000)	2.542	(0.000)	2.578	(0.000)
Own outcome			4.536	(0.000)			2.808	(0.000)
Others' outcomes			1.850	(0.000)			0.299	(0.000)
Equality			−0.216	(0.000)			0.000	(1.000)
*W1* * own outcome			3.241	(0.001)			−0.166	(0.782)
*W2* * others' outcomes			0.247	(0.618)			0.307	(0.024)
*W3* * equality			0.506	(0.003)			−0.235	(0.052)
*Random effects*								
Session	0.241		0.285		0.000		0.000	
Decision maker	0.952		0.841		0.618		0.424	
Directed dyad	0.405		0.178		0.743		0.515	
Number of sessions	16		16		16		16	
Number of decision makers	202		202		200		200	
Number of directed dyads	2874		2874		1892		1892	
Number of decisions	14514		14514		8867		8867	
Log likelihood	−6210.83		−5981.53		−4738.71		−4363.51	

The table shows the hierarchical four-level logistic regression estimates on whether (1) or not (0) a relation is present after an evaluation moment in the *COALOW* and the *COAHIGH* conditions. Definitions of variables are as in Table 2. Random effects are estimated at the level of the session, the decision maker, and the directed dyad under consideration. Numbers of sessions, decision makers, directed dyads, and decisions are given, as are the log likelihoods. *P*-values are in parentheses.

In short, as with the connections model, Hypothesis 1 regarding a positive effect of own outcomes is consistently supported. Now, Hypothesis 2 is also supported: relations that increase others' outcomes are more likely to form. Subjects seem to desire relations that increase inequality or to be indifferent with regards to inequality, in contrast with Hypothesis 3. The evidence for social preferences is mixed (Hypotheses 4 through 6). In the *COALOW* condition, we find support for Hypotheses 4 and 6 regarding the concern for own outcome and equality. In contrast, we only find support for Hypothesis 5 regarding the weight given to others' outcomes in the *COAHIGH* condition. Again, behavior is not or hardly affected by social preferences although we do find a strong and consistent main effect of others' outcomes. Because this effect is not consistently moderated by the individual measurement of social preferences, it cannot be ruled out that this behavioral pattern emerges through a mechanism distinct from intrinsic social preferences. Overall, we thus do not find consistent evidence for effects of social preferences on the network formation decisions in this experiment. Below, we elaborate on the robustness of our results and some alternative explanations for the effects of the social preference variables reported above.

### Robustness analyses

At this stage, it is important to investigate the robustness of our results to some of the assumptions in our analyses. In particular, we assumed an evaluation time of one second after a decision to create or remove a link. Here we investigate whether lengthening the evaluation time influences our results. Furthermore, when a link was created and both subjects “decided” to keep the link we assumed that the subject making the initial decision was the crucial decision maker, because this subject would be more likely to actively evaluate the situation before and after the creation of a relationship. Here we investigate whether altering this assumption, by designating the subjects winning least (or losing most) from the relationship as the crucial decision maker, influences our results. Finally, we assumed that subjects computed other's outcomes and equality on the basis of all subjects in the network. Here we investigate whether assuming that subjects frame more narrowly, either only considering those with whom they have a relationship or the particular subjects with whom they are currently considering a relationship, affects the results. Table 3 depicts the estimates under these alternative assumptions.

**Table 3:** Robustness analyses of logistic regression results.

	Main		Alternative evaluation moment								Alternative crucial actor		Alternative reference group			
			2 sec		3 sec		4 sec		5 sec				Network		Link	
A. *CONLOW*																
Own outcome	3.806	(0.000)	3.979	(0.000)	3.959	(0.000)	3.878	(0.000)	3.832	(0.000)	2.730	(0.000)	3.807	(0.000)	3.697	(0.000)
Others' outcomes	0.242	(0.011)	0.165	(0.076)	0.154	(0.096)	0.132	(0.151)	0.121	(0.183)	1.568	(0.000)	0.420	(0.000)	0.216	(0.000)
Equality	−0.202	(0.000)	−0.200	(0.000)	−0.209	(0.000)	−0.216	(0.000)	−0.220	(0.000)	−0.176	(0.000)	−0.156	(0.000)	−0.025	(0.000)
*W1* * own outcome	0.986	(0.001)	0.533	(0.072)	0.312	(0.297)	0.286	(0.333)	0.355	(0.226)	0.984	(0.001)	0.928	(0.001)	1.067	(0.000)
*W2* * others' outcomes	−0.941	(0.001)	−1.094	(0.000)	−1.092	(0.000)	−1.193	(0.000)	−1.223	(0.000)	−0.773	(0.003)	−0.545	(0.011)	−0.249	(0.118)
*W3* * equality	0.060	(0.691)	0.175	(0.237)	0.071	(0.628)	0.056	(0.696)	0.131	(0.358)	−0.003	(0.982)	0.111	(0.141)	0.123	(0.001)
Number of decisions	21208		21034		20894		20803		20708		21208		21208		21208	
Log likelihood	−9903.68		−10312.97		−10495.91		−10667.64		−10765.08		−10739.90		−9858.45		−9911.90	
B. *CONHIGH*																
Own outcome	2.917	(0.000)	2.992	(0.000)	2.973	(0.000)	2.945	(0.000)	2.915	(0.000)	1.707	(0.000)	2.867	(0.000)	2.876	(0.000)
Others' outcomes	−0.172	(0.050)	−0.275	(0.001)	−0.324	(0.000)	−0.334	(0.000)	−0.413	(0.000)	1.336	(0.000)	−0.100	(0.087)	−0.109	(0.007)
Equality	−0.165	(0.000)	−0.170	(0.000)	−0.164	(0.000)	−0.171	(0.000)	−0.182	(0.000)	−0.108	(0.000)	−0.073	(0.000)	−0.022	(0.000)
*W1* * own outcome	0.729	(0.001)	0.765	(0.001)	0.753	(0.001)	0.672	(0.003)	0.591	(0.008)	0.564	(0.008)	0.733	(0.001)	0.729	(0.001)
*W2* * others' outcomes	−0.072	(0.767)	0.001	(0.998)	−0.037	(0.877)	0.044	(0.851)	−0.074	(0.753)	−0.066	(0.773)	−0.005	(0.977)	−0.054	(0.685)
*W3* * equality	0.037	(0.792)	0.120	(0.378)	0.145	(0.282)	0.179	(0.177)	0.124	(0.348)	−0.026	(0.840)	0.005	(0.939)	−0.029	(0.283)
Number of decisions	19997		19829		19689		19567		19472		19997		19997		19997	
Log likelihood	−9396.76		−9940.49		−10160.33		−10263.39		−10362.14		−10422.29		−9398.19		−9409.32	
C. *COALOW*																
Own outcome	4.536	(0.000)	4.224	(0.000)	3.895	(0.000)	3.609	(0.000)	3.462	(0.000)	3.707	(0.000)	3.576	(0.000)	2.970	(0.000)
Others' outcomes	1.850	(0.000)	1.763	(0.000)	1.720	(0.000)	1.621	(0.000)	1.563	(0.000)	1.777	(0.000)	0.994	(0.000)	−3.318	(0.000)
Equality	−0.216	(0.000)	−0.167	(0.000)	−0.140	(0.000)	−0.123	(0.000)	−0.107	(0.000)	−0.153	(0.000)	−0.026	(0.003)	−0.043	(0.008)
*W1* * own outcome	3.241	(0.001)	3.028	(0.001)	2.495	(0.004)	2.415	(0.004)	2.351	(0.004)	2.271	(0.009)	3.035	(0.002)	2.654	(0.006)
*W2* * others' outcomes	0.247	(0.618)	0.686	(0.138)	0.608	(0.175)	0.743	(0.095)	0.479	(0.271)	0.344	(0.488)	0.165	(0.796)	−0.459	(0.446)
*W3* * equality	0.506	(0.003)	0.441	(0.005)	0.351	(0.020)	0.287	(0.049)	0.316	(0.028)	0.476	(0.004)	0.105	(0.041)	0.185	(0.060)
Number of decisions	14514		14372		14280		14208		14160		14514		14514		14514	
Log likelihood	−5981.53		−6702.57		−7079.85		−7347.49		−7494.88		−6051.47		−6053.45		−5932.72	
D. *COAHIGH*																
Own outcome	2.808	(0.000)	3.053	(0.000)	3.054	(0.000)	2.984	(0.000)	2.952	(0.000)	1.686	(0.000)	2.672	(0.000)	2.672	(0.000)
Others' outcomes	0.299	(0.000)	0.371	(0.000)	0.376	(0.000)	0.349	(0.000)	0.367	(0.000)	0.323	(0.000)	0.185	(0.013)	0.183	(0.014)
Equality	0.000	(1.000)	−0.016	(0.483)	0.002	(0.914)	−0.006	(0.784)	−0.011	(0.618)	0.089	(0.000)	0.012	(0.073)	0.012	(0.072)
*W1* * own outcome	−0.166	(0.782)	−0.554	(0.360)	−0.902	(0.142)	−0.897	(0.144)	−1.231	(0.049)	−0.075	(0.891)	−0.017	(0.977)	−0.017	(0.977)
*W2* * others' outcomes	0.307	(0.024)	0.269	(0.038)	0.275	(0.035)	0.240	(0.066)	0.350	(0.010)	0.388	(0.009)	0.609	(0.001)	0.596	(0.001)
*W3* * equality	−0.235	(0.052)	−0.228	(0.046)	−0.246	(0.032)	−0.330	(0.004)	−0.344	(0.003)	−0.300	(0.018)	−0.006	(0.875)	−0.006	(0.883)
Number of decisions	8867		8775		8692		8636		8592		8867		8867		8867	
Log likelihood	−4363.51		−4851.48		−4993.94		−5068.34		−5096.59		−4535.35		−4378.21		−4383.27	

The table shows hierarchical four-level logistic regression estimates on whether (1) or not (0) a relation is present after an evaluation moment in the four conditions of our experiment. The first columns depict the main results. Subsequent columns show the results if we employ alternative assumptions in our econometric model. The alternative assumptions concern the length of the evaluation moment (how long do subjects take to evaluate a relationship, original assumption being one second), the way the crucial actor is defined in case two actors decide to keep a link (original definition is the one who initiated the link, alternative definition being the one with the lowest earnings from the link), and the definition of the reference group (original assumption being all others, alternatives are only those to whom one is directly connected (Network) or only the subject with whom one is currently considering a relationship (Link)). Random effects are estimated at the level of the session, the decision maker, and the directed dyad under consideration. To keep the table readable, we only report coefficients relating to the hypotheses, the overall number of decisions, and the log likelihood. Definitions of variables are as in Table 2. *P*-values are in parentheses.

We find that estimates are very stable if we lengthen the evaluation moments, both in terms of the magnitude of coefficients and the degree of statistical significance. Two exceptions occur in the *CONLOW* condition, where the effects of others' outcomes and the interaction between caring about own outcomes in the ring and own outcomes in the network game drop in significance if the evaluation moment is taken to be longer.

Adjusting the crucial decision maker when a link is created and maintained to be the subject who earns least (or loses most) from the relationship, rather than the one who initially made the change, also does not change the main conclusions. We do, however, observe that the coefficient on own outcomes decreases in all cases whereas the coefficient on others' outcomes increases in three of the four conditions (*CONLOW, CONHIGH, COAHIGH*). In the *CONHIGH* condition, it even switches from being significantly negative to being significantly positive. Furthermore, the empirical fit drops considerably in all four conditions after we make this change. These two patterns suggest that the original specification was more accurate and that the alternative assumption causes the “more important” own outcomes to be erroneously included in others' outcomes.

Finally, adjusting the reference group also does not alter the conclusions. If we assume that subjects focus either on the subset of subjects to whom they are connected or simply to the subject with whom they are currently evaluating a connection, we do not come to drastically different conclusions. Overall, patterns found are remarkably similar between different reference group specifications.

### Ordering effects

Next to the robustness to specific assumptions made in the analyses, one can ask whether the order in which subjects faced the different conditions influenced their behavior. Such ordering effects can be interesting as they relate to learning. One major assumption in the theoretical literature and the current analyses is that subjects are myopic, purely responding to current outcomes of relations when making their choices. If this assumption is valid, learning opportunities should have little to no effect on behavior. If subjects show foresight and strive to reach certain network positions in the long run, however, learning might have an effect. As subjects gather their own experiences and observe successful strategies of others, they might acquire a better understanding of how to maximize their long-run earnings.

Table 4 depicts the estimates for each condition depending on whether it was the first, second, third, or fourth condition in the experiment. Panels A and B show the results for the *CONLOW* and *CONHIGH* condition respectively. There seems to be no clear trend if subjects have acquired more experience in these conditions. Overall, the positive effect of own outcomes occurs consistently in both conditions, as does the negative effect of equality. For the other variables, results are less robust. The effect of others' outcomes and the interactions do not show a consistent pattern across the two conditions.

**Table 4:** Logistic regression results on ordering of conditions in experiment.

	Main		Condition 1		Condition 2		Condition 3		Condition 4	
A. *CONLOW*										
Own outcome	3.806	(0.000)	3.667	(0.000)	3.629	(0.000)	3.521	(0.000)	4.923	(0.000)
Others' outcomes	0.242	(0.011)	0.499	(0.019)	0.070	(0.679)	−0.035	(0.850)	0.611	(0.013)
Equality	−0.202	(0.000)	−0.333	(0.000)	−0.236	(0.000)	−0.269	(0.000)	−0.118	(0.078)
*W1* * own outcome	0.986	(0.001)	−0.210	(0.717)	1.648	(0.002)	0.530	(0.367)	2.845	(0.000)
*W2* * others' outcomes	−0.941	(0.001)	−1.651	(0.001)	−0.144	(0.786)	−0.640	(0.313)	−0.208	(0.755)
*W3* * equality	0.060	(0.691)	0.082	(0.820)	0.854	(0.001)	−1.223	(0.000)	−0.024	(0.948)
Number of decisions	21208		5355		6058		5334		4461	
Log likelihood	−9903.68		−2417.19		−2864.73		−2550.78		−1937.92	
B. *CONHIGH*										
Own outcome	2.917	(0.000)	2.227	(0.000)	2.995	(0.000)	3.187	(0.000)	3.199	(0.000)
Others' outcomes	−0.172	(0.050)	0.164	(0.423)	−0.299	(0.057)	0.026	(0.890)	−0.558	(0.001)
Equality	−0.165	(0.000)	−0.262	(0.000)	−0.117	(0.009)	−0.108	(0.029)	−0.221	(0.000)
*W1* * own outcome	0.729	(0.001)	2.259	(0.000)	0.495	(0.192)	1.583	(0.003)	−0.460	(0.321)
*W2* * others' outcomes	−0.072	(0.767)	0.293	(0.738)	0.014	(0.967)	0.643	(0.187)	−0.435	(0.387)
*W3* * equality	0.037	(0.792)	0.596	(0.066)	−0.444	(0.055)	0.270	(0.412)	0.088	(0.762)
Number of decisions	19997		3528		6037		5259		5173	
Log likelihood	−9396.76		−1567.76		−2818.06		−2479.22		−2406.10	
C. *COALOW*										
Own outcome	4.536	(0.000)	4.264	(0.000)	4.531	(0.000)	3.522	(0.000)	4.841	(0.000)
Others' outcomes	1.850	(0.000)	0.862	(0.145)	1.597	(0.000)	2.590	(0.000)	1.778	(0.000)
Equality	−0.216	(0.000)	−0.225	(0.006)	−0.177	(0.002)	−0.326	(0.001)	−0.242	(0.000)
*W1* * own outcome	3.241	(0.001)	0.343	(0.915)	3.002	(0.047)	4.512	(0.110)	3.998	(0.013)
*W2* * others' outcomes	0.247	(0.618)	−0.089	(0.964)	1.376	(0.051)	0.373	(0.848)	−0.766	(0.390)
*W3* * equality	0.506	(0.003)	0.117	(0.864)	0.206	(0.502)	0.897	(0.167)	0.664	(0.004)
Number of decisions	14514		4329		2156		4667		3362	
Log likelihood	−5981.53		−1778.75		−963.03		−1799.69		−1403.10	
D. *COAHIGH*										
Own outcome	2.808	(0.000)	2.906	(0.000)	2.949	(0.000)	2.783	(0.000)	2.570	(0.000)
Others' outcomes	0.299	(0.000)	−0.654	(0.009)	0.509	(0.000)	0.235	(0.004)	0.358	(0.000)
Equality	0.000	(1.000)	0.024	(0.670)	−0.080	(0.153)	−0.043	(0.294)	0.036	(0.486)
*W1* * own outcome	−0.166	(0.782)	2.384	(0.039)	1.501	(0.292)	−0.895	(0.387)	−2.213	(0.092)
*W2* * others' outcomes	0.307	(0.024)	−0.017	(0.969)	0.130	(0.622)	0.311	(0.281)	0.324	(0.156)
*W3* * equality	−0.235	(0.052)	−0.241	(0.519)	−0.394	(0.078)	0.030	(0.880)	−0.160	(0.675)
Number of decisions	8867		2819		1356		3024		1668	
Log likelihood	−4363.51		−1357.37		−536.61		−1571.42		−834.66	

The table shows the hierarchical four-level logistic regression estimates on whether (1) or not (0) a relation is present after an evaluation moment in the four conditions in our experiment. The first columns depict the main results. Subsequent columns show the results if we restrict ourselves to the case where the particular condition was the first, second, third, or fourth condition in the experiment. Random effects are estimated at the level of the session, the decision maker, and the directed dyad under consideration. As in Table 3, we restrict our attention to the social preference coefficients, the overall number of decisions, and the log likelihoods. Definitions are as in Table 2. *P*-values are in parentheses.

With respect to learning, the co-author conditions are arguably more interesting than the connections conditions as one can imagine that subjects who are unfamiliar with network formation games will fail to recognize its social dilemma structure. Having gained some experience with network formation subjects might enable subjects to recognize this structure more readily and adjust their behavior accordingly.

Table 4, Panels C and D report the results for the *COALOW* and *COAHIGH* conditions respectively. As for the connections models, we find a consistent positive effect of own outcomes on behavior. Furthermore, the effect of equality is consistently negative in the *COALOW* condition and consistently absent in the *COAHIGH* condition. For the interaction variables, no clear patterns emerge.

The most interesting pattern, however, concerns others' outcomes. If subjects face a co-author condition as the first condition in the experiment they appear to put no weight on the outcomes of others (*COALOW*) or even give them a negative weight (*COAHIGH*). However, if subjects had previous experience with network formation tasks, they consistently attach a positive weight to others' outcomes. It is important to realize that due to the social dilemma nature of these tasks, others' outcomes are positively related with the subject's *long-term self-interest*, and negatively with the person's *short-term self-interest*. These findings are thus in line with the assumption that subjects need some experience with network formation to foresee what is in their own *long-term self-interest*. This reasoning implies that the positive coefficients on others' outcomes may result from strategic decisions in accordance with *long-term self-interest* rather than pure altruistic preferences. Similar arguments are also found in other more complex experimental settings [35], [71], [72].

Finally, the fact that no clear patterns emerge for the social preference interactions is further evidence that such preferences are not of real importance in any of our conditions.

### Decisions in dyads

Social preferences as measured by the ring game seem to play a role in a wide array of decision tasks, but not in these network formation games. In this section, we investigate whether social preferences, in particular altruistic preferences, do play a role in a subset of decisions in the network that more closely mirror the task in which they were elicited.

Recall that the measurements of social preferences are derived from a simple, two-person context. One subject could decide between outcome distributions for herself and a random other subject. This decision situation does not only put all the focus on one specific other subject, it also makes the decision maker directly responsible for how much the other receives. It turns out that in the *COAHIGH* condition, subjects frequently encounter decisions that mirror these properties. In particular, a considerable part of the decisions in the *COAHIGH* condition concern the choice between staying in a mutually exclusive dyad—i.e., two actors who only have a relation with each other and no relations with other actors—and creating an additional relation. Forming an additional relation when one is in a dyad dramatically decreases the outcomes of the dyadic partner by 20 points per minute. In contrast, one's own outcomes decrease only marginally, depending on the number of relations of the subject with whom one is creating this additional relation (see Figure 4). Similar to the *ring game*, the dyadic state likely creates a focus on the outcomes of one particular other, namely the dyadic partner, and the subject is directly responsible for the partner's outcomes. Despite the fact that the subject's own earnings decrease by the creation of an additional link, subject might make such a choice due to error, in the hope that they can maneuver themselves in a more profitable position in the long run, because they put a negative weight on the outcome of the partner, or because they have a preference for outperforming someone else. However, the more subjects value outcomes of others in two-person situations, the less likely they should be to create such relations.

We employ the same pairwise comparison analysis as before, with two exceptions. First, we only look at situations where the pairwise comparison comes down to comparing the option of being in a mutually exclusive dyad with the option to add another relation in the *COAHIGH* condition. Second, we assume that subjects focus on the dyadic partner when assessing the social consequences of the decision. Table 5 displays the results. Note that this analysis includes only a subset of potential variables. In particular, we are unable to estimate a main effect for the weight given to the partner's outcome, as the partner's outcomes always differ by 20 points when comparing the situation with the link to the situation without the link. The interaction effect between partner's outcome and altruistic preferences can be estimated, however, because subjects with different social preferences can put different weight on this 20-point outcome reduction. Next to this, we completely neglect the effect of equality in the current model. The reason being that since the change in the partner's outcome is constant, the difference in equality with or without the link is simply a non-linear transformation of the effect that the link has on the subject's own outcomes. This creates a very strong correlation (Pearson correlation coefficient of −0.977) between own outcomes and equality.

**Table 5:** Logistic regression results for dyadic decisions in the *COAHIGH* condition.

	COAHIGH dyadic decisions			
	Model 1e		Model 2e	
*Fixed effects*				
Constant	−3.035	(0.000)	−2.591	(0.000)
Creation of relation	2.667	(0.000)	2.681	(0.000)
Own outcome			0.901	(0.000)
*W1* * own outcome			0.659	(0.466)
*W2* * partner's outcome			7.050	(0.001)
*Random effects*				
Session	0.000		0.000	
Decision maker	0.846		0.796	
Directed dyad	0.000		0.000	
Number of sessions	16		16	
Number of decision makers	162		162	
Number of directed dyads	545		545	
Number of decisions	1426		1426	
Log likelihood	−571.42		−554.51	

The table shows the hierarchical four-level logistic regression estimates on whether (1) or not (0) a relation is present after an evaluation moment in the *COAHIGH* conditions. We only look at situations where the pairwise comparison comes down to comparing the option of being in a mutually exclusive dyad with the option to add another link. We assume that subjects focus on the dyadic partner when assessing the social consequences of the link. Random effects are estimated at the level of the session, the decision maker, and the directed dyad under consideration. Note that this analysis includes only a subset of potential variables. In particular, the main effect for the weight given to the partner's outcome is omitted as it is constant at -20 by construction. Furthermore, we omit the variables relating to equality. Otherwise, definitions are as in Table 2. *P*-values are in parentheses.

Consistent with earlier analyses, Table 5 shows that subjects are more likely to keep or maintain a link if this link increases their own outcomes. Now, however, we do not find that putting a larger weight on own outcomes in the ring game significantly influences the weight given to the own outcomes in the network formation tasks. In sharp contrast to our other findings, however, we find a significant effect for social preferences regarding the partner's outcomes: subjects that give a higher weight to others' outcomes in the ring game attach a significantly higher weight to the outcome reduction of the dyadic partner. Further analyses, not reported here, indicate that this effect is stable over different lengths of the evaluation period and for using the alternative assumption regarding the crucial decision maker. This suggests that social preferences as measured by the ring game matter during network formation if subjects find themselves in a situation in which their actions have a strong impact on another subject for whom they have a reason to feel responsible. Also, this provides evidence that we have valid social preferences measurements and, therefore, that the lack of effects found in the full set of decisions has a substantive interpretation.

## Conclusion and Discussion

Empirical evidence suggests that people purposively build their social network in order to attain favorable outcomes. This idea has been formalized in theoretical models on network formation, which typically assume myopic self-interest on the side of the actors. Myopic implies that actors only care about the direct consequences of their decisions, while self-interest implies that actors only care about their own outcomes. Experimental evidence suggests that such theories predict network structures well when the outcomes are equal over all actors, but that the predicted networks are seldom formed if they provide unequal outcomes over the actors involved [28], [29]. As these experiments closely match the theoretical environment, this suggests that one needs to relax the micro-level assumption of either myopia or pure self-interest in order to improve the macro-level predictions. Given the large literature on social preferences, and the complexity involved when one wants to relax the assumption of myopia, it may come as no surprise that the most advocated strategy is to relax the assumption of self-interest [28]–[31]. In the current paper, we report on an experiment designed to systematically investigate the role of social preferences in network formation. In particular, we investigated whether behavior in network formation can be related to independent measures of an individual's social preferences.

We developed an experiment in which subjects interacted anonymously in network formation games. We set up this experiment in a way as to maximize our ability to analyze individual decisions. In our experiment, (1) subjects could make changes continuously and received continuous feedback on what the others were doing, (2) there were direct monetary incentives to forming and removing links in the network, and (3) groups were large in size.

Subjects formed networks in four different conditions. As predicted, subjects were found to be more likely to create and maintain relations that increase their own outcomes in the network in each of the conditions. When it comes to altruism results were less clear. We only found consistent evidence for the claim that others' outcomes influence network decisions in the two co-author conditions. In these conditions, others' outcomes are positively related with *long-term self-interest*. Therefore, the positive effect of others' outcomes potentially indicates that subjects foresee what is in their *long-term self-interest*, rather than altruism. This claim is supported by the fact that the positive effect of others' outcomes does not show up in conditions at the beginning of the experiment, when recognizing what is in one's own long-term self-interest is arguably more difficult. Earlier experiments have also found that farsighted behavior often has to develop in the course of the experiment [35]. This suggests that relaxing the assumption of myopia in order to improve macro-level predictions should not be ruled out a priori.

With respect to equality, our estimates suggest that subjects strived for inequality. The argument that people strive for inequality, however, seems unlikely considering the past findings on interdependent decision-making. It is important to note that the effect only consistently appeared in the connections conditions. In these settings, self-interested behavior (possibly combined with a concern for altruism) leads to unequal networks. We cannot completely rule out that the negative effect of equality appears because we are unable to perfectly control for self-interest. Next to this, as subjects do not appear to act in a myopic fashion in the co-author conditions, we cannot rule out that subjects might also use more complex decisions rules in the connections model. As our empirical model assumes myopic decision-making, such alternative decision rules might bias our estimates: what seems to be an effect of a preference for inequality might be an unintended consequence of more complex decision rules. Still, if the argument that subjects have a strong preference for networks that provide equal outcomes was correct, we would expect it to trump these other considerations. It did not, and therefore we can take the findings as reasonable arguments against a prominent role of a preference for equality in these network formation conditions.

We measured social preferences outside a network context by allowing people to choose between sets of own-other outcome distributions. When we relate the behavior in the network to measures of social preferences, we see that they barely ever have an effect; social preferences never contribute substantially to the explained variance. The only finding that occurs somewhat consistently is that those who care more about their own outcomes in the ring game seem to care more about their own outcomes in the network as well. This is not to say that social preferences do not play a role in network formation at all. When we selected cases in which the network formation choice came down to a two-person decision task in which one subject's choice had a considerable effect on the outcomes of a focal other subject, we did find social preferences to be an important predictor of behavior. This result suggests that the measurements of social preferences derived from choices over sets of own-other outcome distributions can predict behavior in similar contexts, but will not straightforwardly generalize to more complex network formation settings. Below, we sketch three explanations for this discrepancy.

First, the complexity of the situation might make it too difficult for subjects to determine which choice is most in line with their social preferences. In a two-person setting, or the multi-person setting that is provided by a public goods game, one's own choices have straightforward consequences for the others; either positive or negative. In a network this is not the case, which makes it more difficult for a person to assess whether a choice is in line with her personal preferences. What should one do if one cares for others' outcomes, but the outcomes for some increase while those of others decrease? Or if one cares about equality but the equality between some increases while the inequality between others decreases? Such considerations are complex and might hamper the role of social preferences in a network context. This complexity is even greater if subjects are not entirely myopic, because the decision task is much more complex in this case.

Second, the complexity might provide subjects with an excuse to alleviate feelings of responsibility. It has been found that people are less likely to display pro-social behavior if they can shift the responsibility for an outcome to third parties [73]. In a network, a number of actors influence the results. Even if a subject would try to increase others' outcomes or equality, it is likely that the choices of others will undo this, either intentionally or unintentionally. Realizing this, actors may choose to strictly focus on their own interest.

Finally, the complexity might trigger a different decision-making process. It has been shown that intuitive decision-making fosters cooperative choices, whereas greater reflection undermines cooperative impulses [74]. The increased complexity that networks provide may lead subjects to employ greater reflection as compared to simple settings, decreasing the importance of social preferences.

As a note on the experimental design, it is important to stress that our experiment focused on income streams rather than accumulated earnings as a relevant criterion to which social preferences relate. Indeed, it would be very difficult if not impossible for our subjects to keep a reasonable count of overall outcomes for all or several subjects in the experiment. We chose this approach as it is in line with the way that social preferences are often employed in experiments and the way in which network formation experiments have been conducted in the past. Future research, however, should investigate the importance of this assumption for our results by allowing subjects to view running averages of outcomes in the course of network formation.

With regard to broader implications, we find that social preferences do not play a major role in the complex process of network formation. Interestingly, we do find evidence suggesting that subjects are able to learn what is in their own long-term self-interest and to decide accordingly. These two results suggest that in order to improve macro-predictions of game-theoretic models of network formation, extending the micro-foundations to include some form of farsightedness may be a more promising direction than the inclusion of social preferences, at least in some contexts. While social preferences do not seem to play a role in the majority of decisions in our networks, we do find social preferences operate when the decision degenerates to a two-person decision task in which one is solely responsible for the outcome of one other person. For many relational decisions in the real world, we can imagine that people feel such a responsibility towards specific others. It would be interesting to systematically investigate under which conditions people frame situations in such a way that makes social preferences relevant, and in which conditions they do not.

## Supporting Information

File S1 **Experimental Instructions.**
(DOC)
