# Supplementary material for: Individual Choices in Dynamic Networks: An Experiment on Social Preferences
Source: PLoS One. 2014 Apr 14;9(4):e92276. doi: 10.1371/journal.pone.0092276 (PMC3986077; doi:10.1371/journal.pone.0092276)
Supplement: File S1 — Experimental Instructions. (DOC) [file pone.0092276.s001.doc]

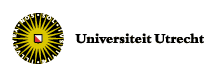
**Experimental Laboratory for Sociology and Economics**

# Scientific Sociological Experiment

# -Instructions-

You are participating in a sociological experiment. Please read the following instructions carefully. These instructions state everything you need to know in order to participate in the experiment. If you have any questions. Please raise your hand. One of the experimenters will approach you in order to answer your question.

You can earn money by means of earning points during the experiment. The number of points that you earn depends on your own choices, and the choices of other participants. At the end of the experiment, the total number of points that you earn during the experiment will be exchanged at an exchange rate of:

**100 points = 1 euro**

The money you earn will be paid out in cash at the end of the experiment. Further instructions on this will follow in due time. During the experiment you are not allowed to communicate with other participants. Also, you may only use the functions on the screen that are necessary for the functioning of the experiment.

**-Overview Experiment-**

The experiment consists of four scenarios of two rounds each, after which you will be asked to participate in some additional decision making situations and to fill in a questionnaire. You find information and explanation of the different scenarios and the decision making situations in this manual. In the four scenarios you and the other participants will be displayed on the screen. At the beginning of each round the positions will be randomly determined. The participants that takes a certain position on the screen in the first round will thus (highly likely) be displayed at another position on the screen in the next round. During the experiment you are displayed as a **blue** hexagon, while the other participants are displayed as **black** dots (see Figure 1).

Each round lasts five minutes. During these five minutes you can earn **points** by creating ties to other participants in the network. Starting from an empty network (see Figure 1) you can let other participants know that you want to create a tie with them by clicking once on the particular participant (see Figure 2). A thin, blue arrow from you to the other participant will appear. Other participants also have the ability to let you know that they want to create a tie with you, if they do so a thin, blue arrow from the other participant towards you will appear.

**Figure 1: Starting situation at the beginning of each round (the number of participants might differ)**


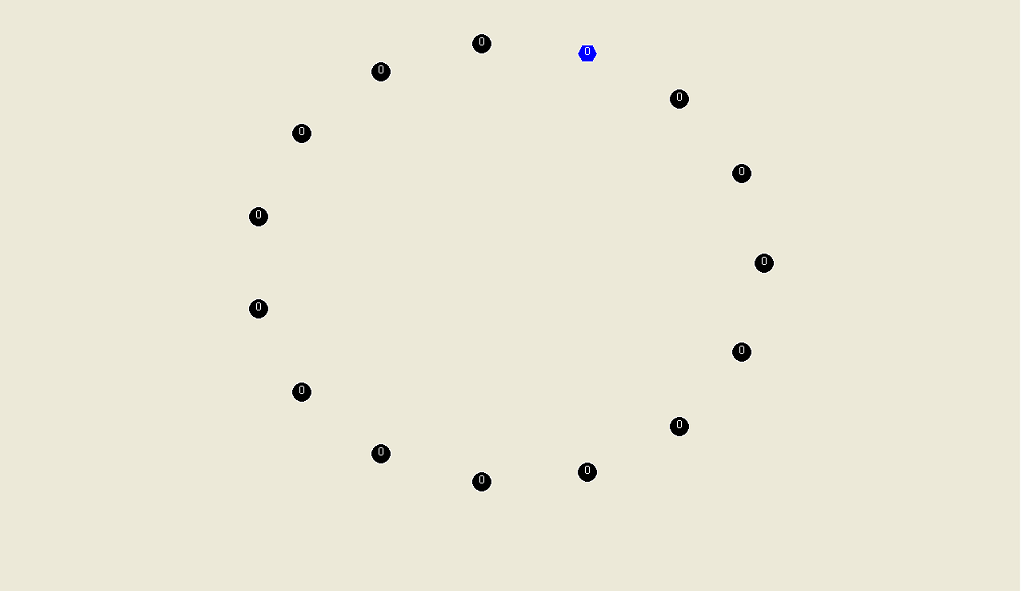


This is you

**Figure 2: Click on one of the other participants in order to show that you want to create a tie with this participant, or to remove an existing tie.**


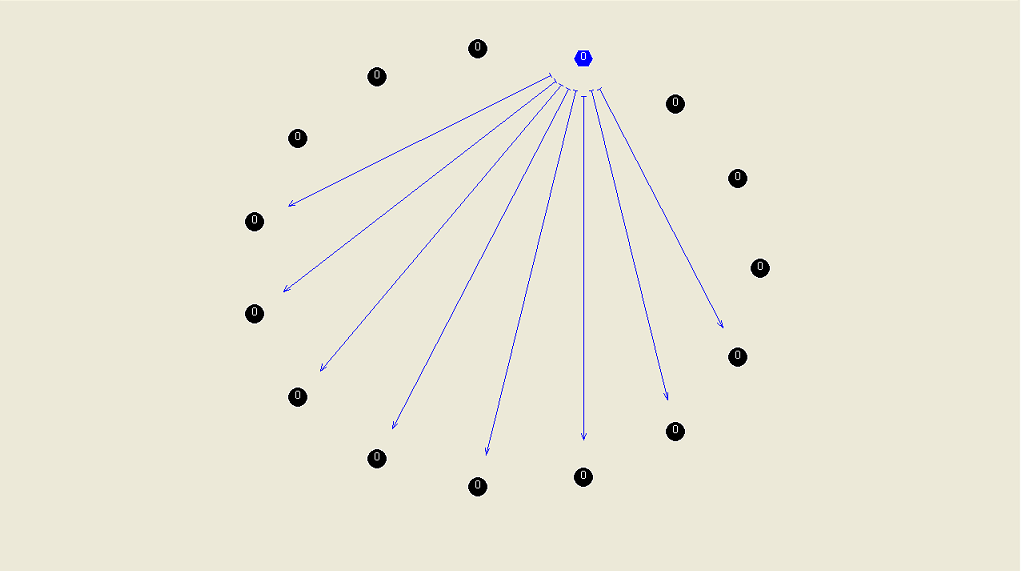


This is you

Click on the other participants in order to propose, create or remove ties

You are only really tied to another participant if the other also acknowledges a desire to create a tie with you. If this is the case, the thin, one-sided arrow will turn into a **thick** two-sided arrow (see Figure 3). If you no longer want a connection with a particular participant you can remove your proposal or tie by clicking on this participants. The rules are the same for all participants in the network. In Figure 3 you see how the situation between two participants can look.

**Figure 3: Tie or no tie? An overview of what you will see on your screen.**

| ***No tie*** |  |
| --- | --- |
| Both you (**blue** hexagon) as well as the other participant (**black** dot), do not show interest in the creation of a tie. | 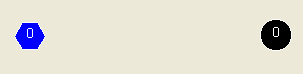 |
| You propose a tie to the other participant, showing that you are interested in creating a tie to him or her. This is however (not yet) a mutual interest. You can withdraw your proposal by clicking on this participant another time. | 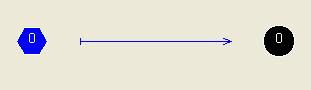 |
| The other participant proposes a tie to you, making clear to you that he or she wants to construct a tie to you. If you want, you can create the tie with this participant by clicking once on the participant. | 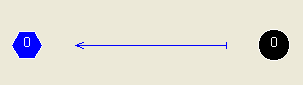 |
| ***Tie*** |  |
| You and another participant both have shown to want a tie with each other. The tie forms and is shown as a **thick, blue** colored, double headed arrow. You can remove the tie by clicking on this participant another time.  Ties between other participants in which you are not involved appear **thick** and colored **black** on the screen**.** | 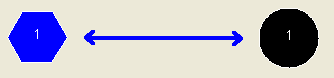  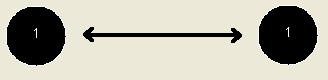 |

**Remarks:**

- It can occur that there is a time-lag between the click and the appearance or disappearance of an arrow on the screen. One click on the participant you want to create, propose or remove a tie to is sufficient: a second click can undo the first click.

**Therefore wait until the arrow appears or disappears before clicking further and making other changes!**

- You only see the proposals for ties that you make yourself or that are made towards you. If other participants make proposals for ties toward each other you will not see this, unless the tie indeed forms.
- You cannot form a tie with yourself or have direct influence on ties between two other participants. With all other participants, you can only form one tie each.
- Please raise your hand if you have questions during the experiment or do not understand something, an experimenter will approach you in order to answer your question.

Depending on the particular scenario, you can earn a certain amount of points *per minute* as a result of the network position that you maintain. The number of points *per minute* that you earn is shown at the bottom of the screen and in the **blue** hexagon. The number of points that the other participants earn are shown in the **black** dots. Next to this, the size of both your hexagon and the dots change with the points that you and the other participants earn: larger in size meaning that the particular participants earn more points per minute. Although the points are *per minute*, they are calculated *per second*. If you earn 90 points for 10 seconds, you receive 10/60 times 90 = 15 points. At the end of each scenario the total number of points that you have earned will be shown.

**-Scenario 1-**

Below it is explained how the number of points that you earn in this scenario depend on the ties that you create. Read this carefully and try to understand the situation. Do not worry if you find it difficult to grasp the situation based on the description below. On the next page two example networks are shown including calculation of points, which might make it more clear. Next to this, there will be a trail round in which you can gain experience in the setting without financial consequences.

The number of points you can earn in this scenario depends on two factors: 1) the benefits of direct connections (i.e. ties), and 2) the benefits of indirect connections through an intermediary.

**Benefits of direct connections:** Each direct connection (i.e. each tie) earns you 1 point per minute.

**Benefits of indirect connections:** Each participant with whom you do not have a tie, but are connected to through an intermediary earns you five points per minute.

It is important to note that each participant with whom you are connected only counts once in the calculation of points, and that the shortest distance is the crucial factor. If you have a direct connection (i.e. a tie) with a participant, then for this tie you receive 1 point per minute, even if you are also indirectly connected to this participant through an intermediary. A participant to whom you have an indirect connection, through an intermediary, earns you 5 points per minute, independent of how many indirect connections you have to this participant. The number of points that a participant earns can be written as follows:

***1 × the number of direct connections (i.e. ties) +***

***5 × the number of participants to whom one is only connected with indirectly through***

***an intermediary***

On the next page two examples are shown of networks and how many points the participants in the networks earn.

**Examples:**


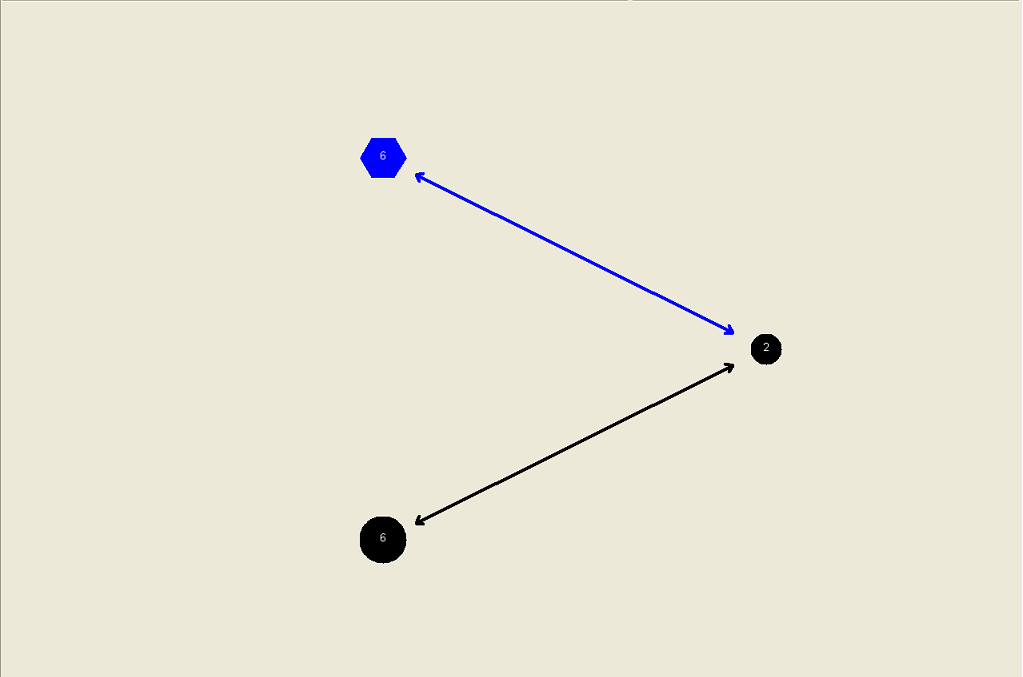


These participants have 1 direct connection (to the third participant) and are indirectly connected to each other through this third participant. Therefore they earn: 1 + 5 = 6 points.

This participant has 2 direct connections with others and therefore earns:

2 × 1 = 2 points.


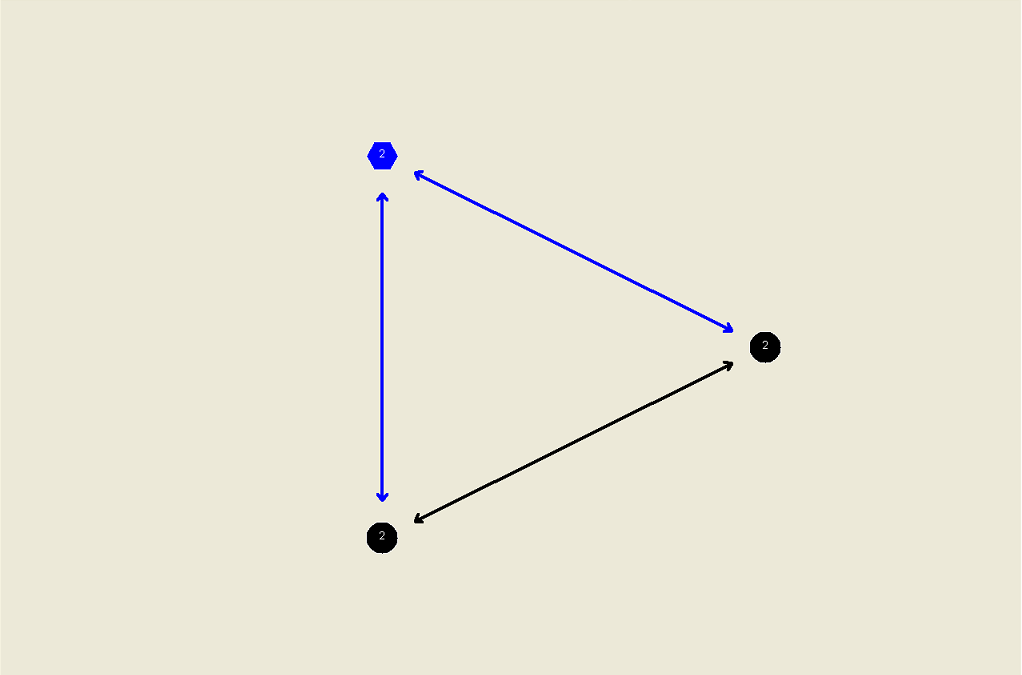


Each participant is directly connected to the other participants. They all earn

2 × 1 = 2 points.

The fact that they are also all indirectly connected to each other does not influence the number of points they earn, because in case of a direct connection only that connection counts.

**Important:** Before the scenario really starts, there will be a trail round of 1.5 minute, in which you can practice with this scenario. In this trail round you cannot earn points. After the trail round, the two “real” rounds in which you can earn points will start. These rounds last for 5 minutes each.

**Don’t turn the page until this scenario is ended...**

**-Scenario 2-**

Below it is explained how the number of points that you earn in this scenario depend on the ties that you create. Read this carefully and try to understand the situation. Do not worry if you find it difficult to grasp the situation based on the description below. On the next page two example networks are shown including calculation of points, which might make it more clear. Next to this, there will be a trail round in which you can gain experience in the setting without financial consequences.

The number of points you can earn in this scenario depends on two factors: 1) the costs of direct connections (i.e. ties), and 2) the benefits of indirect connections through an intermediary.

**Benefits of direct connections:** Each direct connection (i.e. each tie) costs you 1 point per minute.

**Benefits of indirect connections:** Each participant with whom you do not have a tie, but are connected to through an intermediary earns you five points per minute.

It is important to note that each participant with whom you are connected only counts once in the calculation of points, and that the shortest distance is the crucial factor. If you have a direct connection (i.e. a tie) with a participant, then for this tie you lose 1 point per minute, even if you are also indirectly connected to this participant through an intermediary. A participant to whom you have an indirect connection, through an intermediary, earns you 5 points per minute, independent of how many indirect connections you have to this participant. In order to prevent that you will easily lose points, you receive five points per minutes extra. The number of points that a participant earns can be written as follows:

***5 – 1 × the number of direct connections (i.e. ties) +***

***5 × the number of participants to whom one is only connected with indirectly through***

***an intermediary***

On the next page two examples are shown of networks and how many points the participants in the networks earn.

**Examples:**

This participant has 2 direct connections with others and therefore earns:

5 - 2 × 1 = 3 points.

Notice: this is less than what the participant would earn if he or she would have had no connections at all.


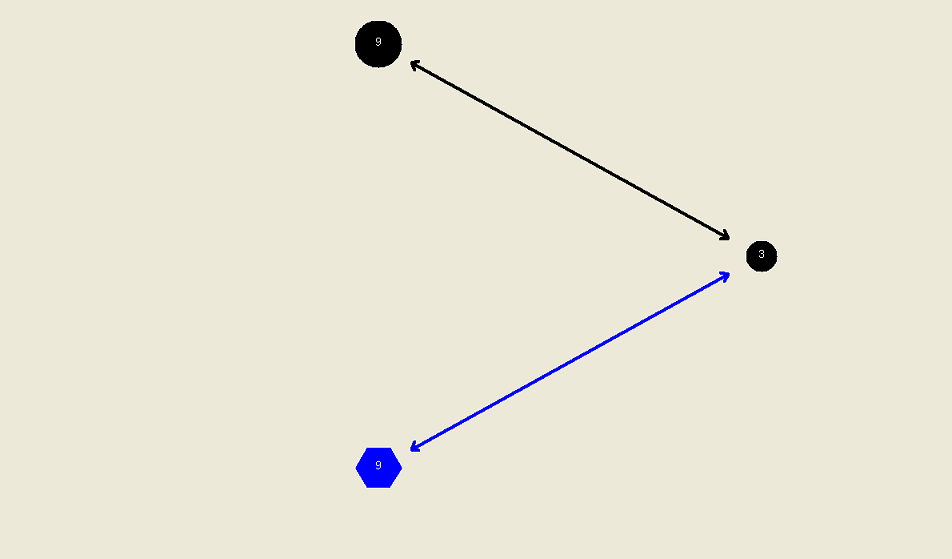


These participants have 1 direct connection (to the third participant) and are indirectly connected to each other through this third participant. Therefore they earn: 5 – 1 + 5 = 9 points.


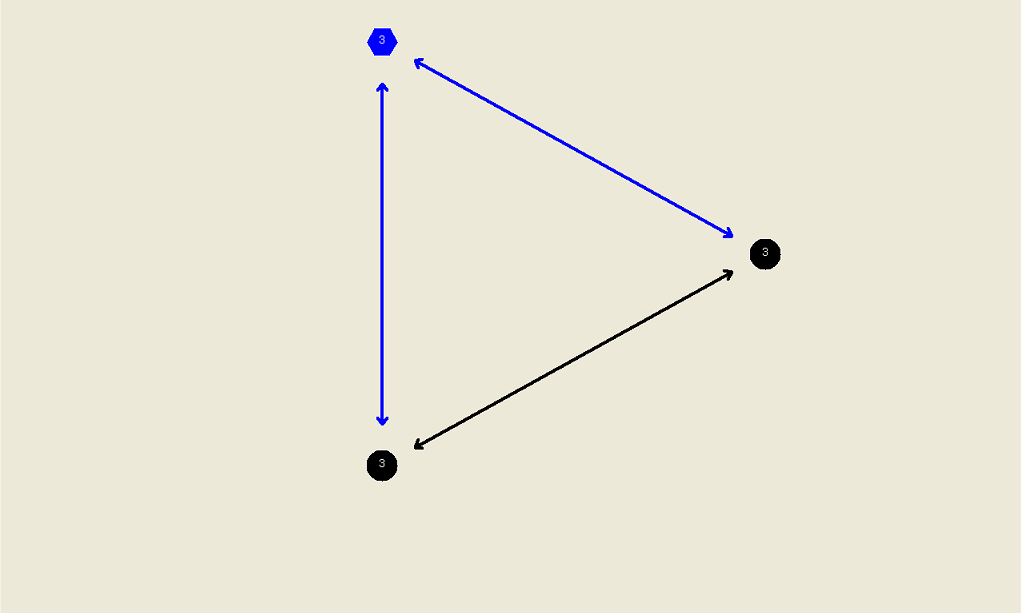


Each participant is directly connected to the other participants. They all earn

5 - 2 × 1 = 3 points.

The fact that they are also all indirectly connected to each other does not influence the number of points they earn, because in case of a direct connection only that connection counts.

Notice: everybody earns less than they would if they would have had no connections at all.

**Important:** Before the scenario really starts, there will be a trail round of 1.5 minute, in which you can practice with this scenario. In this trail round you cannot earn points. After the trail round, the two “real” rounds in which you can earn points will start. These rounds last for 5 minutes each.

**Don’t turn the page until this scenario is ended...**

**-Scenario 3-**

Below it is explained how the number of points that you earn in this scenario depend on the ties that you create. Read this carefully and try to understand the situation. Do not worry if you find it difficult to grasp the situation based on the description below. On the next page two example networks are shown including calculation of points, which might make it more clear. Next to this, there will be a trail round in which you can gain experience in the setting without financial consequences.

The number of points that you can earn in this scenario depends on two factors: 1) the number of ties that you have, 2) the number of ties of the participants to whom you are tied. If we call the number of ties that you have *n_i_*, and the number of ties that a participant with whom you are tied has *n_j_*, for each tie you have you receive:

$20\cdot\left(\frac{1}{n_{i}}+\frac{1}{n_{j}}+\frac{1}{n_{i}\cdot n_{j}}\right)$ points.

Because the above calculation is hard to execute, we provide an overview of the returns of a tie given the number of ties that you and the other participant have. In order to keep the table somewhat manageable, we do not provide all possible combinations. An important principle that is clearly observable in the table is that a tie to a participant returns more points if the other participant has few connections.

|  |  | **The number of ties you have** | | | | | | | |
| --- | --- | --- | --- | --- | --- | --- | --- | --- | --- |
| **The number of ties that a participant with whom you are tied has** |  | **1** | **2** | **3** | **4** | **5** | **7** | **10** | **15** |
|  | **1** | 60 | 40 | 33 | 30 | 28 | 26 | 24 | 23 |
|  | **2** | 40 | 25 | 20 | 18 | 16 | 14 | 13 | 12 |
|  | **3** | 33 | 20 | 16 | 13 | 12 | 10 | 9 | 8 |
|  | **4** | 30 | 18 | 13 | 11 | 10 | 9 | 8 | 7 |
|  | **5** | 28 | 16 | 12 | 10 | 9 | 7 | 6 | 6 |
|  | **7** | 26 | 14 | 10 | 9 | 7 | 6 | 5 | 4 |
|  | **10** | 24 | 13 | 9 | 8 | 6 | 5 | 4 | 3 |
|  | **15** | 23 | 12 | 8 | 7 | 6 | 4 | 3 | 3 |

Using the table above, you can calculate your total number of points and calculate or estimate how this changes if you create another tie. For example, if you have 4 ties with 3 participants who each have 1 tie, and 1 participant who has 2 ties, you earn
3 × 30 + 1 × 18 = 108 points. Returns that are not shown in the table, for instance if you have 6 ties, are neatly placed between the values that are shown. Notice that if you create a new tie, this decreases the value of existing ties.

On the next page two examples are shown of networks and how many points the participants in the networks earn.

**Examples:**

**
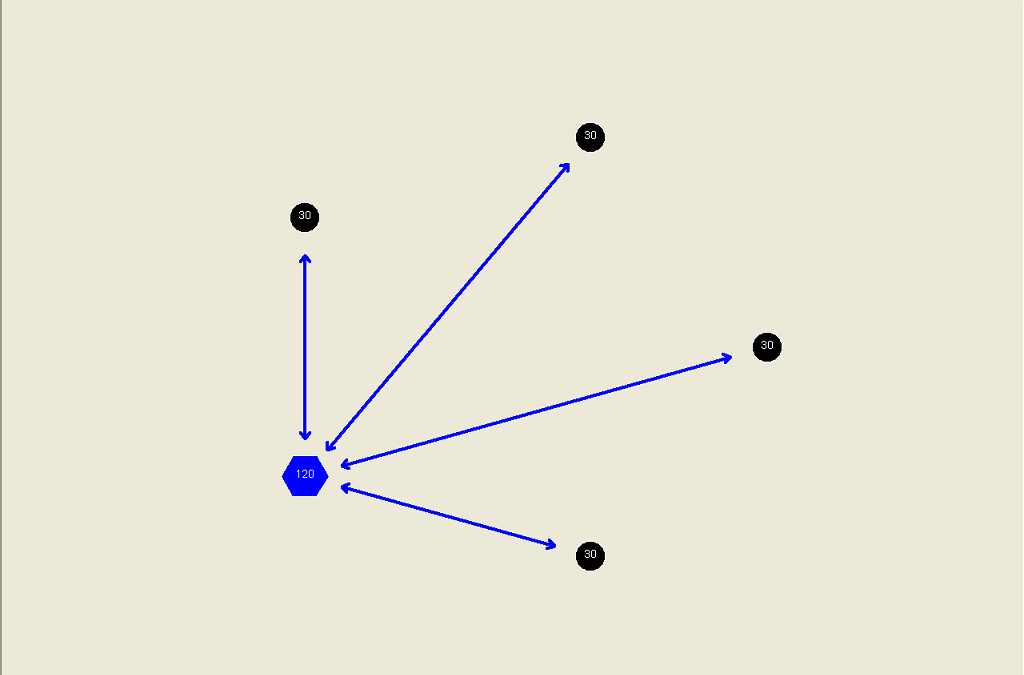
**

The blue colored participant has 4 ties to participants who each have 1 tie. Therefore, this participant earns:

4 × 30 = 120 points

These 4 participants all have 1 tie with a participant who has 4 ties. Therefore, these participants earn 30 points.

**
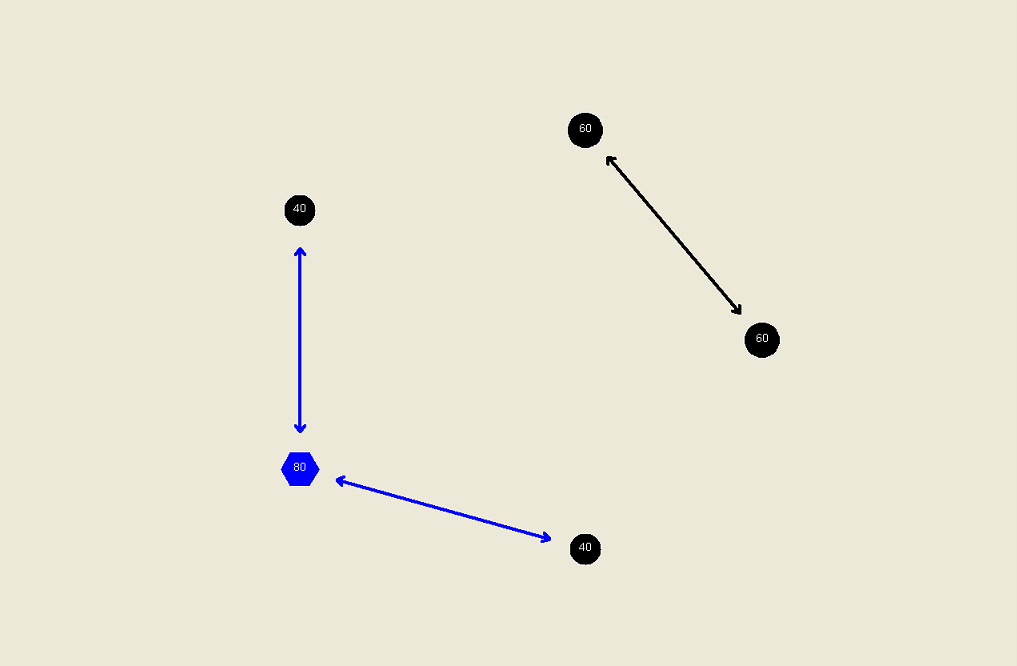
**

This participant has 2 ties, with participants who have 1 tie each. The participants therefore earn:

2 × 40 = 80 points.

These 2 participants both have 1 tie with another participant who has 2 ties. They therefore earn 40 points.

These two participant each have 1 tie with another participant who also has 1 tie. They therefore earn 60 points.

**Important:** Before the scenario really starts, there will be a trail round of 1.5 minute, in which you can practice with this scenario. In this trail round you cannot earn points. After the trail round, the two “real” rounds in which you can earn points will start. These rounds last for 5 minutes each.

**Don’t turn the page until this scenario is ended...**

**-Scenario 4-**

Below it is explained how the number of points that you earn in this scenario depend on the ties that you create. Read this carefully and try to understand the situation. Do not worry if you find it difficult to grasp the situation based on the description below. On the next page two example networks are shown including calculation of points, which might make it more clear. Next to this, there will be a trail round in which you can gain experience in the setting without financial consequences.

The number of points that you can earn in this scenario depends on two factors: 1) the number of ties that you have, 2) the number of ties of the participants to whom you are tied. If we call the number of ties that you have *n_i_*, and the number of ties that a participant with whom you are tied has *n_j_*, for each tie you have you receive:

$20\cdot\left(\frac{1}{n_{i}}+\frac{1}{n_{j}}+\frac{1}{n_{i}\cdot n_{j}}\right)-6$ points.

Because the above calculation is hard to execute, we provide an overview of the returns of a tie given the number of ties that you and the other participant have. In order to keep the table somewhat manageable, we do not provide all possible combinations. An important principle that is clearly observable in the table is that a tie to a participant returns more points if the other participant has few connections.

|  |  | **The number of ties you have** | | | | | | | |
| --- | --- | --- | --- | --- | --- | --- | --- | --- | --- |
| **The number of ties that a participant with whom you are tied has** |  | **1** | **2** | **3** | **4** | **5** | **7** | **10** | **15** |
|  | **1** | 54 | 34 | 27 | 24 | 22 | 20 | 18 | 17 |
|  | **2** | 34 | 19 | 14 | 12 | 10 | 8 | 7 | 6 |
|  | **3** | 27 | 14 | 10 | 7 | 6 | 4 | 3 | 2 |
|  | **4** | 24 | 12 | 7 | 5 | 4 | 3 | 2 | 1 |
|  | **5** | 22 | 10 | 6 | 4 | 3 | 1 | 0 | 0 |
|  | **7** | 20 | 8 | 4 | 3 | 1 | 0 | -1 | -2 |
|  | **10** | 18 | 7 | 3 | 2 | 0 | -1 | -2 | -3 |
|  | **15** | 17 | 6 | 3 | 1 | 0 | -2 | -2 | -3 |

Using the table above, you can calculate your total number of points and calculate or estimate how this changes if you create another tie. For example, if you have 4 ties with 3 participants who each have 1 tie, and 1 participant who has 2 ties, you earn
3 × 24 + 1 × 12 = 84 points. Returns that are not shown in the table, for instance if you have 6 ties, are neatly placed between the values that are shown. Notice that if you create a new tie, this decreases the value of existing ties.

On the next page two examples are shown of networks and how many points the participants in the networks earn.

**Examples:**

**
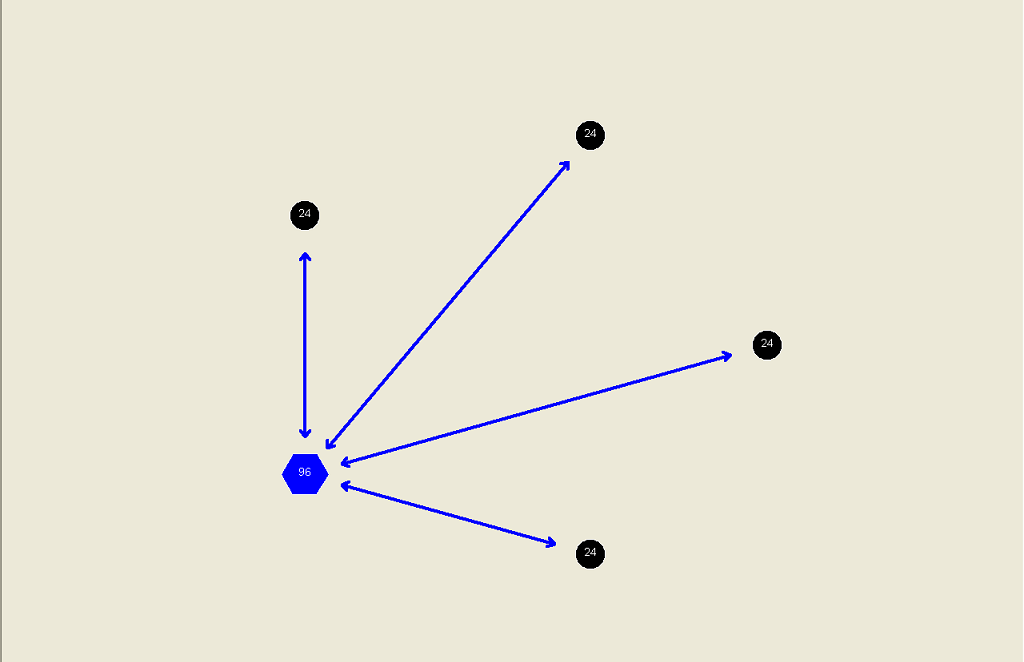
**

The blue colored participant has 4 ties to participants who each have 1 tie. Therefore, this participant earns:

4 × 24 = 96 points.

These 4 participants all have 1 tie with a participant who has 4 ties. Therefore, these participants earn 24 points.

**
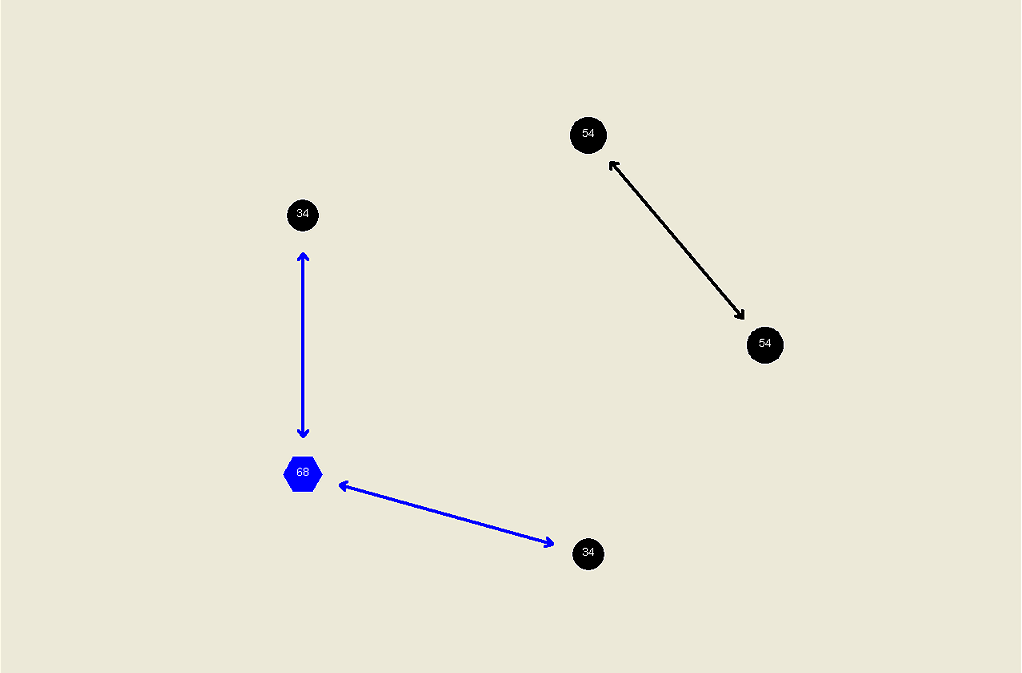
**

This participant has 2 ties, with participants who have 1 tie each. The participants therefore earn:

2 × 34 = 68 points.

These 2 participants both have 1 tie with another participant who has 2 ties. They therefore earn 34 points.

These two participant each have 1 tie with another participant who also has 1 tie. They therefore earn 54 points.

**Important:** Before the scenario really starts, there will be a trail round of 1.5 minute, in which you can practice with this scenario. In this trail round you cannot earn points. After the trail round, the two “real” rounds in which you can earn points will start. These rounds last for 5 minutes each.

**Don’t turn the page until this scenario is ended...**

**-Decision situation-**

You will be presented twenty-four decision situations. In each of these situations you get to choose between two options, option A and option B. Both options vary in terms of the amount of points that you earn and the amount of points that will be allocated to a randomly selected participant who is currently present in the laboratory. These points will be added to the earnings of the earlier scenarios. If you choose option A in the screen displayed below, than you receive 10 points, and a randomly selected other participant receives 20 points. If you choose option B than you receive 7.4 points, and the other participant receives 19.7 points. Who the “other participant” is, is determined before each decision situation is presented by means of a random lottery procedure. The other participant is not shown how much he/she receives as a result of your choice. At the end of the twenty-four decision situations the points for each participant will be calculated and added to the total.


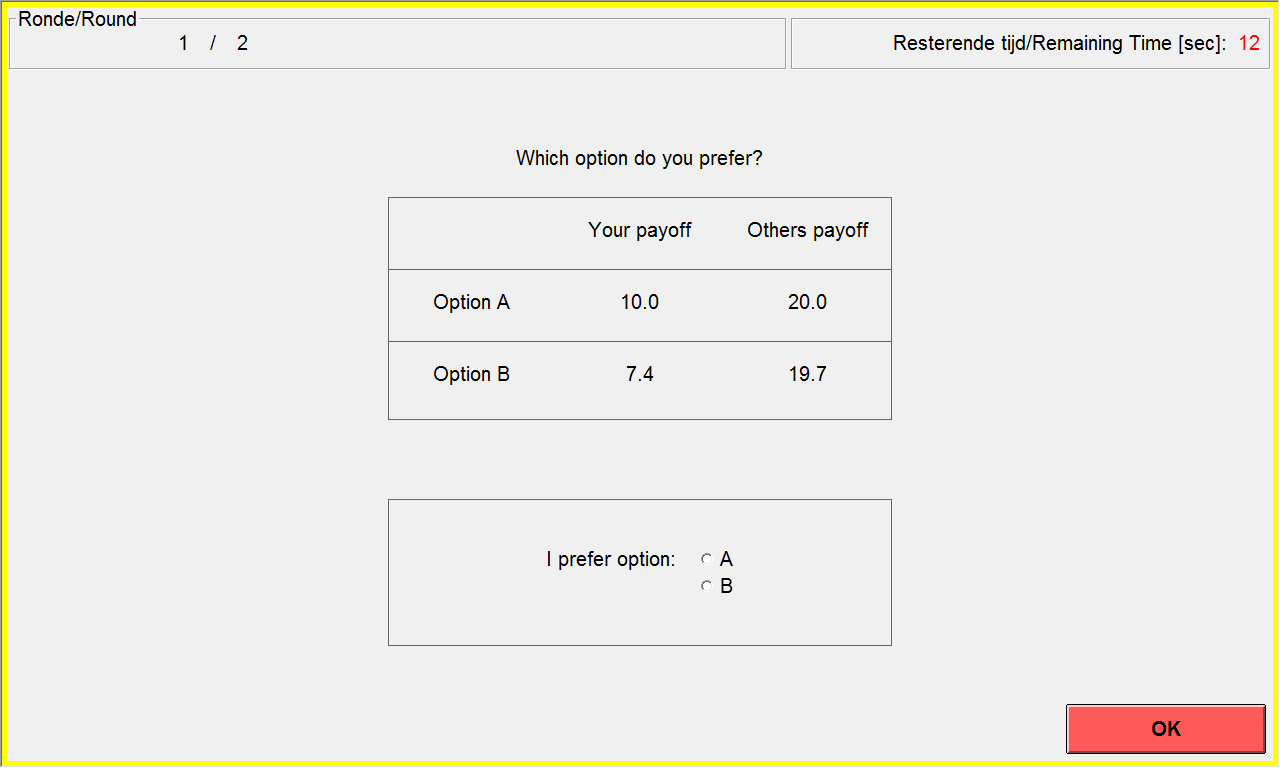


**After these decision situations you will be asked to fill in a questionnaire. Please take your time to fill in this questionnaire accurately. In the mean time your earnings will be counted. Please remain at your seat until the payment has taken place.**
